# On the bromination of the dihydroazulene/vinylheptafulvene photo-/thermoswitch

**DOI:** 10.3762/bjoc.8.108

**Published:** 2012-06-27

**Authors:** Virginia Mazzanti, Martina Cacciarini, Søren L Broman, Christian R Parker, Magnus Schau-Magnussen, Andrew D Bond, Mogens B Nielsen

**Affiliations:** 1 Department of Chemistry, University of Copenhagen, Universitetsparken 5, DK-2100 Copenhagen Ø, Denmark; 2 Sino-Danish Center for Education and Research (SDC), Niels Jensens Vej 2, DK-8000 Aarhus C, Denmark; 3 Department of Chemistry, University of Florence, via della Lastruccia 3-13, I-50019, Sesto F.no, Italy; 4 Department of Physics, Chemistry and Pharmacy, University of Southern Denmark, Campusvej 55, DK-5230 Odense M, Denmark

**Keywords:** azulene, bromination, dihydroazulene, molecular switches, photoswitch, vinylheptafulvene

## Abstract

**Background:** The dihydroazulene (DHA)/vinylheptafulvene (VHF) system (with two cyano groups at C1) functions as a photo-/thermoswitch. Direct ionic bromination of DHA has previously furnished a regioselective route to a 7,8-dibromide, which by elimination was converted to a 7-bromo-substituted DHA. This compound has served as a central building block for functionalization of the DHA by palladium-catalyzed cross-coupling reactions. The current work explores another bromination protocol for achieving the isomeric 3-bromo-DHA and also explores the outcome of additional bromination of this compound as well as of the known 7-bromo-DHA.

**Results:** Radical bromination on two different VHFs by using *N*-bromosuccinimide/benzoyl peroxide and light, followed by a ring-closure reaction generated the corresponding 3-bromo-DHAs, as confirmed in one case by X-ray crystallography. According to a ^1^H NMR spectroscopic study, the ring closure of the brominated VHF seemed to occur readily under the reaction conditions. A subsequent bromination–elimination protocol provided a 3,7-dibromo-DHA. In contrast, treating the known 7-bromo-DHA with bromine generated a very labile species that was converted to a new 3,7-dibromoazulene, i.e., the fully unsaturated species. Azulenes were also found to form from brominated compounds when left standing for a long time in the solid state. Kinetics measurements reveal that the 3-bromo substituent enhances the rate of the thermal conversion of the VHF to DHA, which is opposite to the effect exerted by a bromo substituent in the seven-membered ring.

**Conclusion:** Two general procedures for functionalizing the DHA core with a bromo substituent (at positions 3 and 7, respectively) are now available with the DHA as starting material.

## Introduction

**Scheme 1:**
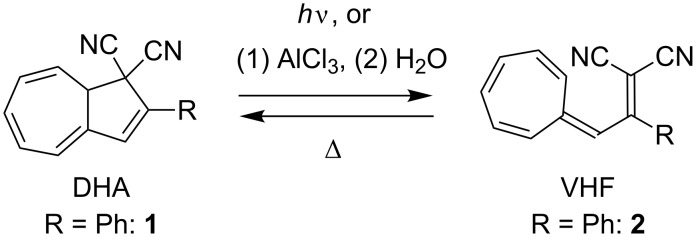
Dihydroazulene (DHA)/vinylheptafulvene (VHF) photo-/thermoswitch.

1,8a-Dihydroazulene-1,1-dicarbonitrile (DHA, **1**) is a yellow photochromic compound, which undergoes a light-induced 10-electron retro-electrocyclization to a red-colored vinylheptafulvene (VHF) (Scheme 1) [1–3]. The VHF compound is formed as the s-*cis* conformer, which, however, is in equilibrium with the more stable s-*trans* conformer. The s-*cis* VHF undergoes a thermally induced cyclization to regenerate the original DHA. The significant structural difference between the DHA and VHF forms, as reflected in their different colors and hence electronic properties, renders the system interesting as a light-controlled molecular switch in, for example, molecular electronics. Indeed, light-induced conductance switching was recently observed for a DHA derivative situated in a single-molecule junction [4]. For the further exploration of the DHA/VHF switch in this field, ongoing synthetic efforts are required for the incorporation of functional groups onto the system, especially in a regioselective manner.

**Figure 1:**
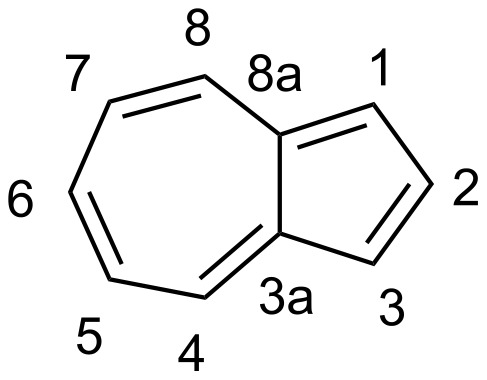
Numbering of azulene.

**Scheme 2:**
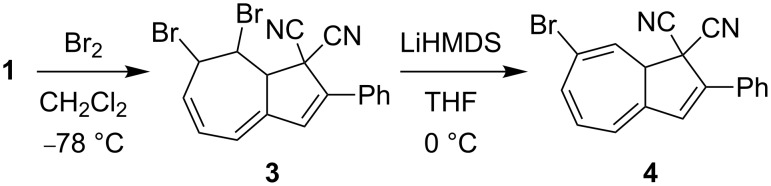
Bromination–elimination protocol for functionalization of DHA [3,5–8]. HMDS = hexamethyldisilazide.

**Scheme 3:**
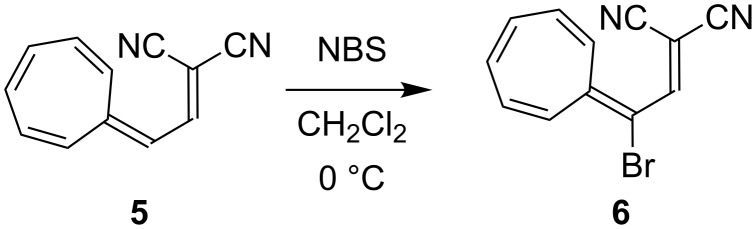
Bromination of VHF [11]. NBS = *N*-bromosuccinimide.

We have recently developed an efficient protocol for functionalizing the DHA/VHF **1**/**2** (R = Ph) at position 7 (for numbering, see Figure 1) by a bromination–elimination protocol of **1**, as shown in Scheme 2 [3,5–8]. Elimination of HBr from the intermediate **3** provided the bromo-functionalized DHA **4** that was employed for further cross-coupling reactions. The advantage of this method is that the “parent DHA” **1** can be prepared on a large scale in a few steps [9] and it is hence a convenient building block for further functionalization. Moreover, we have found that the treatment of DHA by aluminum chloride followed by water provided another means of inducing ring-opening of DHA to form VHF (Scheme 1) [10]. This method is particularly convenient for making VHF on a preparative scale, which is more tedious when employing a light source. Along this line, we became interested in investigating the possibility of brominating the VHF **2**. It was previously shown by Kuroda and Asao [11] that the related VHF **5** underwent bromination by *N*-bromosuccinimide (NBS) to form the product **6** (Scheme 3), but any thermal conversion of this VHF to a DHA was not described. Here we describe NBS bromination of VHF **2** and isolation of the corresponding 3-bromo-DHA. In addition, the outcome of further bromination of this compound as well as of the 7-bromo-DHA **4** is presented.

## Results and Discussion

### Synthesis

**Scheme 4:**
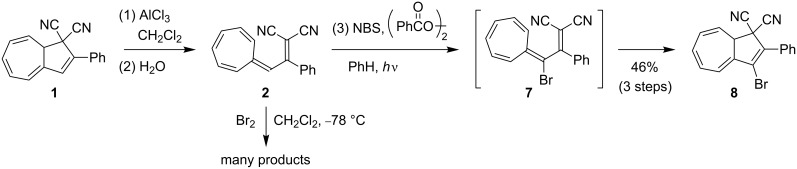
Radical and ionic brominations of VHF.

**Figure 2:**
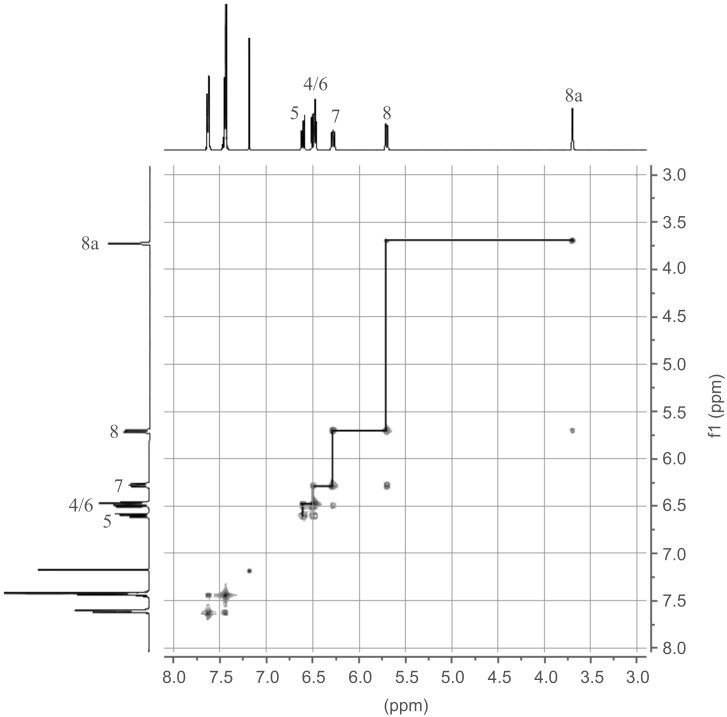
^1^H,^1^H COSY NMR spectrum of DHA **8** (CDCl_3_, 500 MHz). For assignments of DHA signals, see numbering in Figure 1.

**Figure 3:**
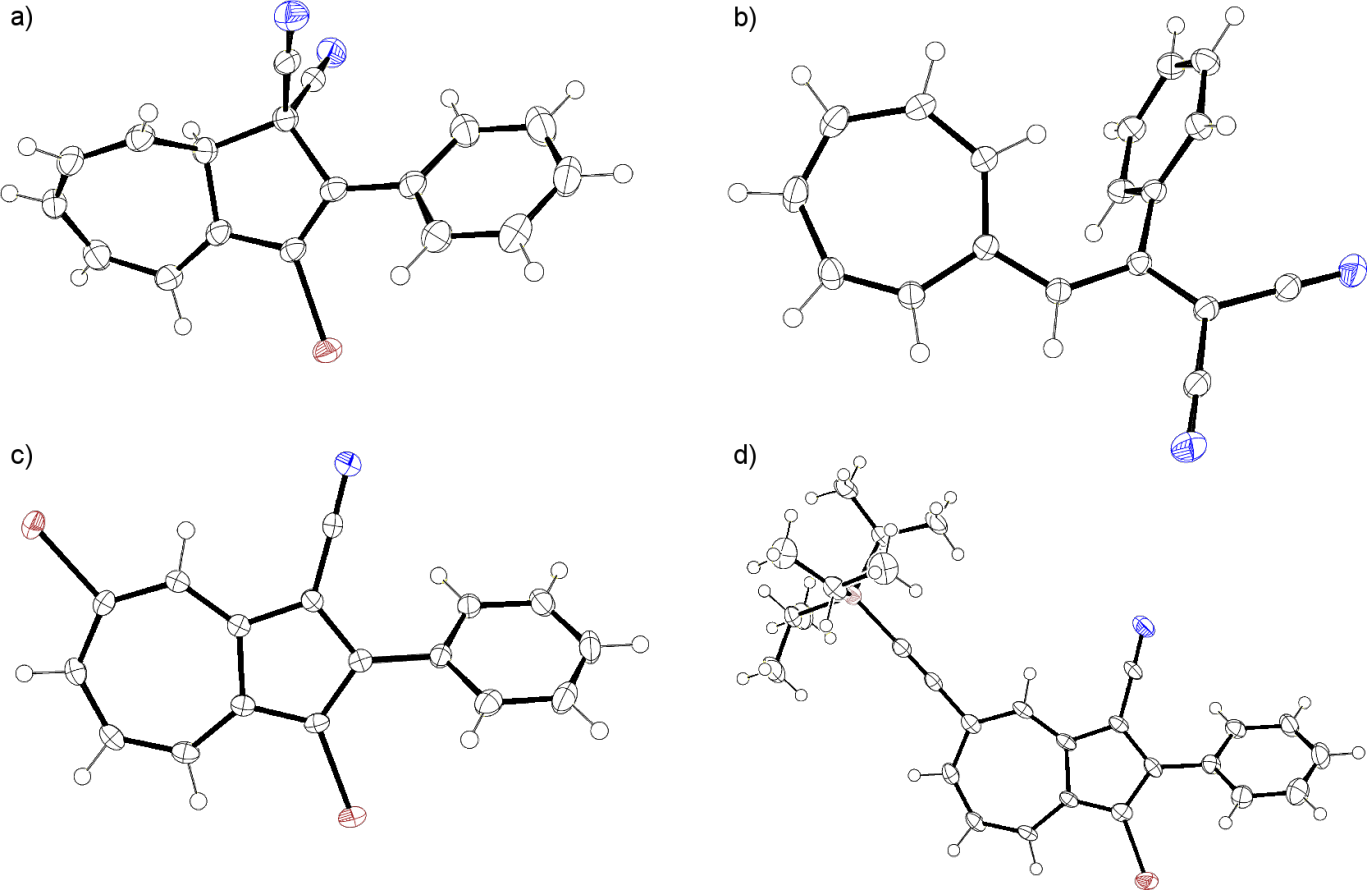
Molecular structures (with displacements ellipsoids at 50% probability for non-H atoms) of (a) DHA **8** (CCDC 868717; crystals were grown from butanone/heptane); (b) VHF **2** (CCDC 866016; crystals were grown from benzene/cyclohexane; benzene cocrystallized with the VHF but has been omitted for clarity); (c) azulene **14** (CCDC 868718; crystals were grown from dichloromethane/heptane); (d) azulene **15** (CCDC 868719; crystals were grown from dichloromethane/heptane).

**Figure 4:**
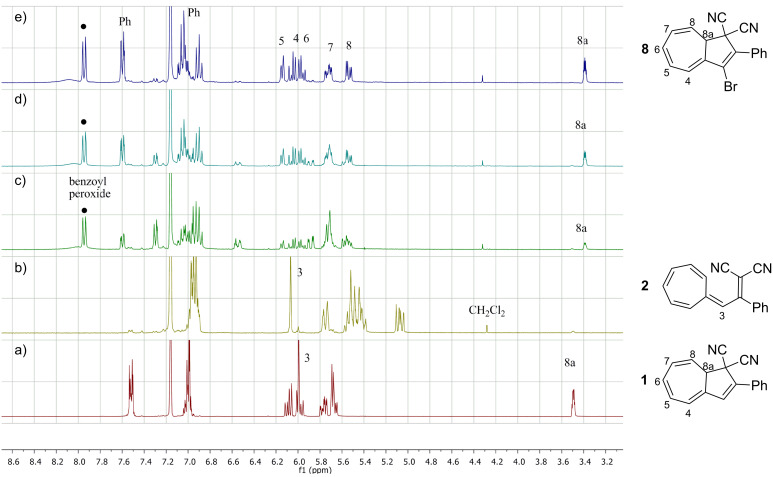
^1^H NMR spectra (C_6_D_6_, 300 MHz) of (a) DHA **1**; (b) VHF **2**; (c–e) VHF **2** after treatment with 2 molar equiv of NBS in the presence of benzoyl peroxide and irradiation for 5, 15, and 35 min, respectively.

DHA **1** was first opened to VHF **2** by aluminum chloride followed by quenching with water according to a previously described procedure (Scheme 4) [10]. The resulting VHF **2** was then treated with NBS and benzoyl peroxide in benzene and the mixture was subjected to irradiation from a 500 W halogen lamp source, presumably generating the intermediate, but short-lived (see below), species **7**. No structural evidence for **7** was obtained, but after standard work-up, the ring-closed 3-bromo-DHA product **8** was isolated in an overall yield of 46%, suggesting that **7** is indeed formed as an intermediate. The structure of **8** was elucidated by a ^1^H,^1^H COSY NMR spectrum (Figure 2) and ultimately confirmed by X-ray crystallographic analysis (Figure 3a). Interestingly, when following the bromination by ^1^H NMR spectroscopy, signals from the VHF **2** seem absent within 5 min, while instead signals from the DHA **8** quickly emerge (Figure 4). There are, however, some signals around 6.5 and 7.3 ppm (Figure 4c and Figure 4d) from unidentified intermediates, which decrease in intensity after irradiation for longer time. No characteristic signals from the suggested bromo-VHF intermediate **7** were observed, thus it must undergo rapid ring closure to the DHA **8** under the reaction conditions although the mixture is subjected to light.

**Figure 5:**
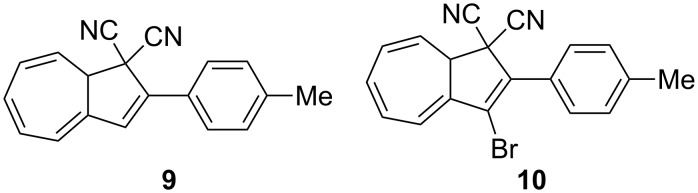
Compound **9** was selectively brominated to furnish the product **10**.

Interestingly, when the known tolyl derivative **9** [12] (Figure 5) was subjected to the ring opening followed by NBS bromination, the 3-bromo-DHA **10** was formed in almost quantitative yield (estimated yield of 95%) instead of the benzylic bromide product. This compound was, however, difficult to purify without significant loss of material. When subjecting VHF **2** to bromination by Br_2_ (Scheme 4), under the same conditions used to regioselectively brominate the corresponding DHA **1**, many products (unidentified) were formed according to NMR spectroscopy. Thus, ionic bromination is not a useful method for the functionalization of the VHF. Gratifyingly, we managed to grow crystals suitable for X-ray diffraction studies of the VHF **2** (generated by the AlCl_3_-induced ring opening of DHA **1** followed by a liquid–liquid extraction) by layering cyclohexane upon a solution of the VHF in benzene and allowing this mixture to crystallize at 5 °C (lower temperatures and nonpolar solvents reduce the rate of the undesired thermal back reaction [9]). The crystal structure of VHF **2** is shown in Figure 3b.

**Scheme 5:**
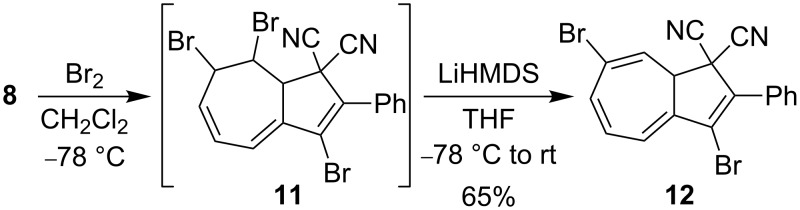
Synthesis of 3,7-dibromo-DHA.

**Scheme 6:**
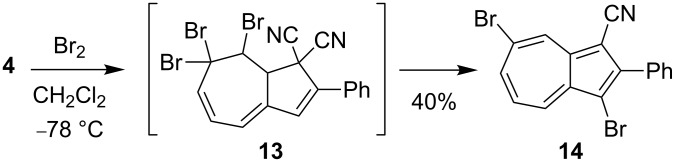
Synthesis of a 3,7-dibromoazulene.

With the objective to generate DHA building blocks with more than one bromo functionality for further reactions, we subjected the 3-bromo-DHA **8** to the ionic bromination–elimination protocol, which, via the intermediate **11**, provided the 3,7-dibromo-substituted DHA **12** (Scheme 5). In an alternative strategy (Scheme 6), we started out with the 7-bromo-DHA **4**. Treatment with bromine at −78 °C generated in this case, however, a very labile intermediate, tentatively assigned to the structure **13**, which underwent ready conversion, without the addition of base, to the azulene **14** together with a complex mixture of other nonisolated products. This product is not surprising, inasmuch as we have previously found that a solution of the related dibromide **3** over time underwent conversion to a mixture of 1-bromo-3-cyano-2-phenylazulene and 1-cyano-2-phenylazulene [5]. The conversion of **13** into azulenes was, however, so fast that we could not perform the controlled elimination of HBr by LiHMDS to generate the corresponding 7-bromo-DHA as we could from **3**. The structure of the azulene **14** was confirmed by X-ray crystallographic analysis (Figure 3c). Functionalized azulenes are themselves interesting in materials chemistry for their optical and redox properties [13].

**Scheme 7:**
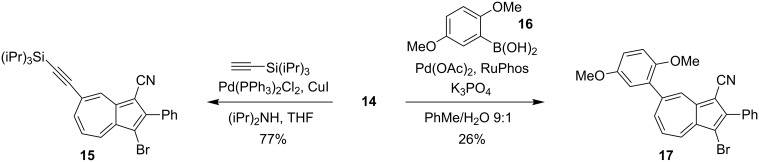
Regioselective Sonogashira and Suzuki couplings. RuPhos = 2-dicyclohexylphosphino-2',6'-diisopropoxy-1,1'-biphenyl.

The two bromo positions of **14** showed very different reactivity. Thus, subjecting **14** to a Sonogashira coupling with triisopropylsilylacetylene by using the Pd(PPh_3_)_2_Cl_2_/CuI catalyst system only gave the monocoupled product **15** (Scheme 7), confirmed by X-ray crystal structure analysis (Figure 3d). Moreover, a Suzuki cross-coupling reaction with boronic acid **16** gave the product **17**, albeit in rather low yield (Scheme 7). The substitution was confirmed by NOESY 1D experiments (see Supporting Information File 1).

**Scheme 8:**
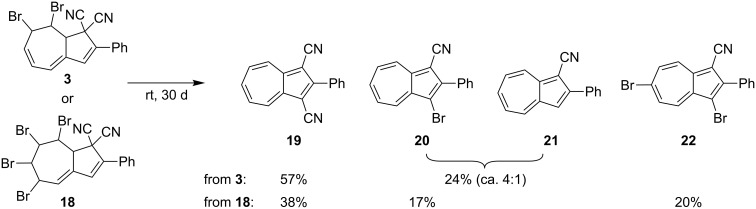
Slow conversions to azulenes in the solid state.

Finally, we observed that upon storage at room temperature, the dibromide **3** as well as the known tetrabromide **18** [5] slowly turned into a mixture of azulenes, which we isolated and identified as **19**–**22** shown in Scheme 8. While, **19** (X-ray crystal structure is given in [6]), **20** [5], and **21** [5,14–15] are already known, the dibromoazulene **22** is new.

### UV–vis absorption and switching studies

**Figure 6:**
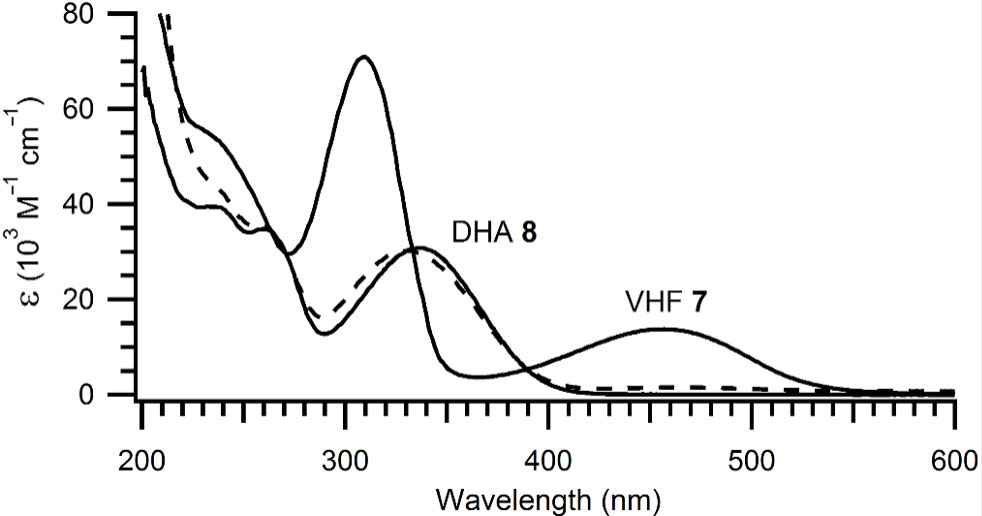
Absorption spectra of DHA **8** and VHF **7** in cyclohexane. The broken curve shows the absorption spectrum after one light–heat cycle (DHA → VHF → DHA).

The UV–vis absorption spectrum of the 3-bromo-DHA **8** in cyclohexane is shown in Figure 6. The compound exhibits an absorption maximum at 337 nm, which is blue-shifted by 17 nm relative to that of DHA **1** [9]. By irradiation at 337 nm, the DHA was gradually converted to the VHF **7** (Figure 6) exhibiting a characteristic absorption maximum at 453 nm (blue-shifted by 7 nm relative to that of VHF **2** [9]). Upon heating of the VHF at 50 °C, a gradual conversion to the DHA was observed. Although both the light-induced DHA-to-VHF conversion and the thermally induced VHF-to-DHA conversion were found to occur with isosbestic points in the absorption spectra, we found that the conversion was not fully reversible. First, the VHF could not be fully converted to DHA (Figure 6, broken curve), and, second, when a second cycle was performed, the final VHF absorption was at a lower intensity than after the first conversion. Nevertheless, the decay of the VHF absorption could be fitted to an exponential decay (see Supporting Information File 1).

From this fit, we obtained rough estimates of the rate constant and half-life of *k* = 5.1 × 10^−4^ s^−1^ and *t*_1/2_ = 27 min at 50 °C. For comparison, the values for conversion of VHF **2** to DHA **1** in cyclohexane are 8.3 × 10^−5^ s^−1^ and *t*_1/2_ = 139 min at 50 °C (found from Arrhenius plot, [9]). Thus, the influence of the bromo substituent in **7** is to enhance significantly the rate of the thermal ring closure, which is explained by its inductive electron withdrawal, hence stabilizing a zwitterionic VHF transition state, as previously suggested [9]. In accordance hereto, we have previously found that moving the bromo substituent to the seven-membered ring has the opposite effect; thus, the VHF derived from 7-bromo-DHA **4** underwent ring closure considerably slower than VHF **2** [7]. We are, however, not able to explain the remarkably fast formation of **8** from **7** under the bromination conditions described above, but it may be the result of the slight Lewis acidity under the reaction conditions.

## Conclusion

In conclusion, we have developed a method for functionalizing the DHA/VHF photo-/thermoswitch with a bromo substituent at position 3 in the DHA core (product **8**). The synthesis explores the ready conversion of DHA **1** to VHF **2** on a preparative scale by using aluminum chloride followed by NBS bromination. This bromination was found to occur selectively in the presence of a tolyl group (product **10**). The method contrasts the earlier bromination (Br_2_)–elimination protocol on DHA for incorporating selectively a bromo substituent at position 7 in the DHA core without proceeding via the VHF as an intermediate. Subjecting the 3-bromo-DHA **8** to this bromination–elimination protocol furnished the 3,7-dibromo-DHA **12**. In contrast, subjecting the 7-bromo-DHA **4** to an additional bromination with Br_2_ generated a very labile compound that was readily converted to the fully unsaturated 3,7-dibromo-substituted azulene **14** together with other unidentified products. The instability of intermediate bromide addition products was also reflected by solid-state conversions of di- and tetrabromides to a variety of azulenes. The influence on the thermal VHF-to-DHA conversion exerted by a bromo substituent at position 3 (DHA numbering) is opposite to that of one at position 7; the former substitution enhances the ring closure while the latter retards it. The new bromo-substituted compounds will be interesting for future scaffolding in the quest for advanced photo- and thermoswitches. We note, however, that initial attempts at using the 3-bromo-substituted DHA for Sonogashira or Suzuki couplings (as previously accomplished for the 7-bromo-substituted DHA) have so far been unsuccessful. It seems that the 3-bromo functionality is not very reactive, which is reflected as well by the regioselective Sonogashira and Suzuki reactions on the 3,7-dibromoazulene **14** reported in this work.

## Experimental

### 

#### General Methods

NMR spectra were measured on 300 or 500 MHz instruments. All chemical shift values in the ^1^H and ^13^C NMR spectra are referenced to the solvent (δ_H_ = 7.26 ppm, δ_C_ = 77.16 ppm). Thin-layer chromatography (TLC) was carried out on commercially available precoated plates (silica 60) with fluorescence indicator; color change of the spot from yellow (DHA) to red (VHF) upon irradiation by UV light indicated the presence of a DHA. For column chromatographic purification of DHAs, the column was covered by aluminium foil to exclude light; the isolated fractions were also kept in the dark. All melting points are uncorrected. All spectroscopic measurements (including photolysis of DHA to VHF and kinetics studies on the thermal conversion of VHF to DHA) were performed in a cuvette of 1 cm path length. Photoswitching experiments were performed by using a 150 W xenon arc lamp equipped with a monochromator. Elemental analyses were performed at the Department of Chemistry, University of Copenhagen.

**3-Bromo-2-phenyl-1,8a-dihydroazulene-1,1-dicarbonitrile (8):** To a stirred solution of DHA **1** (300 mg, 1.17 mmol) in CH_2_Cl_2_ (40 mL) at rt was added AlCl_3_ (800 mg, 6.01 mmol). Stirring was continued for further 20 min and then the mixture was quenched with water (60 mL). The organic layer was separated, dried over anhydrous Na_2_SO_4_, filtered, and concentrated under reduced pressure. The resulting red compound was then dissolved in benzene (40 mL) and NBS (700 mg, 3.93 mmol) was added. The solution was stirred under an Ar atmosphere for 10 min and then benzoyl peroxide (13 mg, 0.05 mmol) was added. The solution was irradiated with a 500 W halogen lamp kept at a distance of ca. 1 m from the reaction mixture. After 4 h, the mixture was filtered and washed with water. The organic layer was washed with saturated aqueous NaHCO_3_ solution (3 × 100 mL), dried over anhydrous Na_2_SO_4_, and filtered. The solvent was removed under reduced pressure and the crude residue was purified by dry column vacuum chromatography (SiO_2_, 60% Et_2_O/heptane) affording **8** (180 mg, 46%) as a yellow solid. *R*_f_ 0.73 (30% heptane/EtOAc); mp 126 °C; ^1^H NMR (500 MHz, CDCl_3_) δ 7.63 (m, 2H), 7.44 (m, 3H), 6.61 (dd, *J* = 11.3, 6.4 Hz, 1H), 6.50 (d, *J* = 6.1 Hz, 1H), 6.49–6.46 (m, 1H), 6.28 (ddd, *J* = 10.1, 6.1, 2.1 Hz, 1H), 5.70 (dd, *J* = 10.1, 4.0 Hz, 1H), 3.70 (m, 1H) ppm; ^13^C NMR (125 MHz, CDCl_3_) δ 136.5, 135.8, 131.8, 130.8, 130.4, 130.3, 129.0, 128.8, 128.10, 126.9, 122.1, 119.6, 114.3, 111.9, 48.8, 47.4 ppm; FABMS *m*/*z*: [M]^+^ 334; Analysis calcd for C_18_H_11_BrN_2_: C, 64.50; H, 3.31; N, 8.36; found: C, 63.90; H, 3.42; N, 7.86.

**3-Bromo-2-(4-methylphenyl)-1,8a-dihydroazulene-1,1-dicarbonitrile (10):** To a stirred solution of 2-(4-methylphenyl)-1,8a-dihydroazulene-1,1-dicarbonitrile (**9**) (250 mg, 0.97 mmol) in CH_2_Cl_2_ (30 mL) at rt was added AlCl_3_ (760 mg, 5.74 mmol). Stirring was continued for a further 10 min and then the mixture was quenched with water (50 mL). The organic layer was separated, dried over Na_2_SO_4_, filtered, and concentrated under reduced pressure. The residue was then dissolved in benzene (50 mL), and NBS (1.60 g, 9.50 mmol) was added. The solution was stirred under an Ar atmosphere for 10 min and then benzoyl peroxide (3 mg, 0.01 mmol) was added. The solution was irradiated with a 500 W halogen lamp for 2 h. The mixture was filtered and washed with water. The organic layer was washed with saturated aqueous NaHCO_3_ solution (3 × 150 mL), dried over anhydrous Na_2_SO_4_, and filtered. The solvent was removed under reduced pressure affording **10** with minor impurities (321 mg, estimated yield of 95%). An analytically pure sample (yellow solid) could be obtained by dry column vacuum chromatography (SiO_2_, Et_2_O/heptane 3:2). *R*_f_ 0.79 (30% ethyl acetate/heptane); mp 115 °C; ^1^H NMR (500 MHz, CDCl_3_) δ 7.62 (d, *J* = 8.0 Hz, 2H), 7.32 (d, *J* = 8.0 Hz, 2H), 6.67 (dd, *J* = 11.3, 6.4 Hz, 1H), 6.58–6.49 (m, 2H), 6.34 (ddd, *J* = 10.1, 6.1, 2.1 Hz, 1H), 5.76 (dd, *J* = 10.1, 4.0 Hz, 1H), 3.79–3.72 (m, 1H), 2.43 (s, 3H) ppm; ^13^C NMR (125 MHz, CDCl_3_) δ 140.8, 136.6, 136.0, 131.6, 130.3, 129.7, 128.6, 128.1, 127.8, 126.2, 121.8, 119.6, 114.4, 112.0, 48.8, 47.3, 21.5 ppm; EIMS *m*/*z*: [M]^+^ 348; Analysis calcd for C_19_H_13_BrN_2_: C, 65.35; H, 3.75; N, 8.02; found: C, 65.50; H, 3.54; N, 7.94.

**3,7-Dibromo-2-phenyl-1,8a-dihydroazulene-1,1-dicarbonitrile (12):** 3-Br-DHA **8** (85 mg, 0.25 mmol) was dissolved in CH_2_Cl_2_ (5 mL) at −78 °C under a N_2_ atmosphere and the mixture was excluded from light. Then a solution of Br_2_ in CH_2_Cl_2_ (0.78 M, 327 µL) was slowly added over 2 min. The mixture was stirred for 30 min at −78 °C and then concentrated in vacuo to yield the crude product **11** as a yellow-brown solid (126 mg, 0.25 mmol). This compound was used without purification for the next step. It was dissolved in dry THF (5 mL) and cooled to −78 °C. Then LiHMDS (0.3 mL, 0.3 mmol, 1 M in toluene) was added dropwise, and the solution was stirred for 2 h, while the temperature was slowly raised to rt. The reaction mixture was diluted with Et_2_O (20 mL) and then washed with saturated aqueous NH_4_Cl (2 × 50 mL). The organic phase was dried over anhydrous Na_2_SO_4_ and filtered. The solvent was removed under reduced pressure and the crude residue was purified by dry column vacuum chromatography (SiO_2_, 50% Et_2_O/heptane) affording **12** (68 mg, 65%) as a pale green solid. Mp 118 °C; ^1^H NMR (300 MHz, CDCl_3_) δ 7.78–7.64 (m, 2H), 7.61–7.46 (m, 3H), 6.63 (d, *J* = 3.6 Hz, 2H), 6.52 (dt, *J* = 3.6, 1.7 Hz, 1H), 6.08 (d, *J* = 4.5 Hz, 1H), 3.80 (dd, *J* = 4.5, 1.7 Hz, 1H) ppm; ^13^C NMR (125 MHz, CDCl_3_) δ 138.3, 137.9, 133.8, 131.3, 130.7, 130.3, 129.1, 128.7, 126.4, 121.2, 120.4, 120.2, 113.7, 111.5, 49.0, 46.9 ppm; Analysis calcd for C_18_H_10_Br_2_N_2_: C, 52.21; H, 2.43; N, 6.76; found: C, 52.58; H, 2.41; N, 6.69.

**3,7-Dibromo-2-phenylazulene-1-carbonitrile (14):** To a solution of **4** excluded from light (154 mg, 0.46 mmol) (prepared as previously described [12]) in CH_2_Cl_2_ (4 mL) at −78 °C under a N_2_ atmosphere was slowly added a 0.78 M solution of Br_2_ (0.59 mL, 0.46 mmol) in CH_2_Cl_2_. After being stirred for 10 min at −78 °C, the reaction mixture was concentrated in vacuo. Purification by flash column chromatography (SiO_2_, 50% CH_2_Cl_2_/heptane) afforded **14** (71 mg, 40%) as a green solid. Mp 220–223 °C; ^1^H NMR (500 MHz, CDCl_3_) δ 8.89 (d, *J* = 2.1 Hz, 1H), 8.52 (d, *J* = 9.9 Hz, 1H), 8.17 (dd, *J* = 10.4, 1.8 Hz, 1H), 7.84 (m, 2H), 7.64–7.49 (m, 3H), 7.38 (t, *J* = 10.4 Hz, 1H) ppm; ^13^C NMR (125 MHz, CDCl_3_) δ 152.5, 143.3, 141.7, 139.5, 139.4, 137.2, 132.7, 130.3, 130.0, 128.9, 127.4, 124.4, 116.3, 105.4, 96.1 ppm; GC–MS (*m/z*): [M]^+^ 386.9; HRMS–ESI (*m*/*z*): [M + H]^+^ calcd for C_17_H_10_Br_2_N, 385.9175; found, 385.9196.

**3-Bromo-2-phenyl-7-(triisopropylsilylethynyl)azulene-1-carbonitrile (15):** To a mixture of the azulene **14** (18 mg, 0.047 mmol), Pd(PPh_3_)_2_Cl_2_ (4 mg) and CuI (2 mg) in argon-degassed THF (4 mL) under an Ar atmosphere were added iPr_2_NH (40 μL, 0.283 mmol) and triisopropylsilylacetylene (30 μL, 0.134 mmol). After being stirred for 24 h at rt, additional triisopropylsilylacetylene (50 μL, 0.223 mmol) was added, and the mixture was stirred again for 24 h at rt. Concentration of the reaction mixture in vacuo and purification of the residue by flash column chromatography (50% CH_2_Cl_2_/heptane) afforded **15** (17.5 mg, 77%) as a green solid. Mp 181–183 °C; ^1^H NMR (500 MHz, CDCl_3_) δ 8.72 (d, *J* = 1.5 Hz, 1H), 8.51 (dd, *J* = 9.9, 0.7 Hz, 1H), 8.03 (ddd, *J* = 10.4, 1.5, 1.0 Hz, 1H), 7.86–7.80 (m, 2H), 7.60–7.56 (m, 2H), 7.54–7.51 (m, 1H), 7.51 (t, *J* = 10.2 Hz, 1H), 1.22–1.16 (m, 21H) ppm; ^13^C NMR (125 MHz, CDCl_3_) δ 151.9, 143.7, 142.1, 139.4, 139.2, 138.0, 132.9, 130.3, 129.8, 128.8, 127.4, 124.1, 116.5, 108.9, 106.0, 97.6, 95.1, 18.9, 11.5 ppm; HRMS–ESI (*m*/*z*): [M + H]^+^ calcd for C_28_H_31_BrNSi, 488.1404; found, 488.1398.

**3-Bromo-7-(2’,5’-dimethoxyphenyl)-2-phenylazulene-1-carbonitrile (17):** To a solution of the azulene **14** (50 mg, 0.129 mmol) and 2,5-dimethoxyphenylboronic acid (**16**) (47 mg, 0.259 mmol) under an Ar atmosphere in an argon-degassed toluene/water 9:1 mixture were added K_3_PO_4_ (110 mg, 0.517 mmol), Pd(OAc)_2_ (6 mg, 0.0026 mmol) and RuPhos (2-dicyclohexylphosphino-2',6'-diisopropoxy-1,1'-biphenyl) (24 mg, 0.051 mmol). The mixture was vigorously stirred and heated at 100 °C for 4 d. Purification by flash column chromatography (SiO_2_, CH_2_Cl_2_) afforded **17** (15 mg, 26%) as a blue solid. Mp 169–171 °C; ^1^H NMR (500 MHz, CDCl_3_) δ 8.81 (d, *J* = 1.7 Hz, 1H), 8.56 (dd, *J* = 10.0, 0.9 Hz, 1H), 8.00 (ddd, *J* = 10.2, 1.7, 0.9 Hz, 1H), 7.86–7.83 (m, 2H), 7.64 (t, *J* = 10.1 Hz, 1H), 7.60–7.55 (m, 2H), 7.53–7.47 (m, 1H), 7.06–6.88 (m, 3H), 3.85 (s, 3H), 3.80 (s, 3H) ppm; ^13^C NMR (125 MHz, CDCl_3_) δ 154.1, 151.0, 150.6, 142.5, 142.4, 139.5, 139.4, 139.3, 137.2, 133.3, 132.9, 130.3, 129.5, 128.8, 127.7, 117.2, 117.1, 114.6, 113.0, 104.2, 96.8, 56.5, 56.1 ppm; HRMS–ESI (*m*/*z*): [M + H]^+^ calcd for C_25_H_19_BrNO_2_, 444.0594; found, 444.0617.

#### Solid-state conversion of dibromide **3** into azulenes (**19**–**21**)

The dibromide **3** (416 mg, 1.00 mmol) was stored in the dark at rt in a closed vessel (50 mL) for 30 d. The resulting black solid was dissolved in CH_2_Cl_2_ and purification by dry column vacuum chromatography (SiO_2_, 12.6 cm^2^, 0–100% toluene/heptanes, 12.5% steps, then 0–35% CH_2_Cl_2_ in toluene, 7% steps, 40 mL fractions) gave **19** (119.4 mg, 57%) and an inseparable mixture (ca. 4:1) of **20** and **21** (55.8 mg, 24%) (known compounds: [5,14–15]).

**2-Phenylazulene-1,3-dicarbonitrile (19):** ^1^H NMR (500 MHz, CDCl_3_) δ 8.80 (d, *J* = 9.7 Hz, 2H), 8.08 (t, *J* = 9.7 Hz, 1H), 8.05 (d, *J* = 7.4 Hz, 2H), 7.87 (t, *J* = 9.7 Hz, 2H), 7.62 (t, *J* = 7.4 Hz, 2H), 7.58–7.55 (m, 1 H) ppm; ^13^C NMR (125 MHz, CDCl_3_) δ 155.6, 145.4, 141.7, 138.1, 131.8, 131.7, 130.9, 129.7, 129.6, 116.1, 97.0 ppm; for X-ray data, see [6].

#### Solid-state conversion of tetrabromide **18** into azulenes **19**, **20**, and **22**

The tetrabromide **18** (576 mg, 1.00 mmol) was stored in the dark at rt in a closed vessel (50 mL) for 30 d. The resulting black solid was dissolved in CHCl_3_ and purification by flash column chromatography (SiO_2_, toluene) gave **19** (78.9 mg, 38%) as a pink solid, **20** (50.8 mg, 17%) as a violet solid, and **22** (97.7 mg, 20%) as a green solid. TLC (toluene); *R*_f_ 0.06–0.09 (**19**), 0.25–0.30 (**20**), 0.43–0.47 (**22**).

**3,6-Dibromo-2-phenylazulene-1-carbonitrile (22):** ^1^H NMR (300 MHz, CDCl_3_) δ 8.32 (d, *J* = 8.2 Hz, 1H), 8.28 (d, *J* = 8.6 Hz, 1H), 7.94 (dd, *J* = 5.2 Hz, *J* = 1.9 Hz, 1H), 7.90 (dd, *J* = 4.6 Hz, *J* = 1.9 Hz, 1H), 7.81–7.85 (m, 2H), 7.49–7.61 (m, 3H) ppm; ^13^C NMR (75 MHz, CDCl_3_) δ 151.3, 142.1, 138.6, 137.9, 136.2, 134.4, 132.8, 132.2, 131.7, 130.2, 129.8, 128.9, 116.2, 106.6, 98.5 ppm; Analysis calcd for C_17_H_9_Br_2_N: C, 52.75; H, 2.34; N, 3.62; found: C, 52.97; H, 2.03; N, 3.55.

## Supporting Information

File 1 1D and 2D NMR spectra of all new compounds. Table of bond lengths for VHF **2** (X-ray crystallographic data). Exponential fit of the decay over time of the VHF **7** absorbance at the longest-wavelength absorption maximum.
