# Supplementary material for: On the bromination of the dihydroazulene/vinylheptafulvene photo-/thermoswitch
Source: Beilstein J Org Chem. 2012 Jun 27;8:958–66. doi: 10.3762/bjoc.8.108 (PMC3388886; doi:10.3762/bjoc.8.108)
Supplement: File 1 — 1D and 2D NMR spectra of all new compounds. Table of bond lengths for VHF 2 (X-ray crystallographic data). Exponential fit of the decay over time of the VHF 7 absorbance at the longest-wavelength absorption maximum. [file Beilstein_J_Org_Chem-08-958-s001.pdf]

Supporting Information

for

On the bromination of the dihydroazulene/vinylheptafulvene photo-/thermoswitch

Virginia Mazzanti[1,2], Martina Cacciarini[1,3], Søren Lindbæk Broman[1], Christian R. Parker[1], Magnus Schau-Magnussen[1], Andrew D. Bond[4] and Mogens Brøndsted Nielsen[1*]

Address: [1]Department of Chemistry, University of Copenhagen, Universitetsparken 5, DK-2100 Copenhagen Ø, Denmark, [2]Sino-Danish Center for Education and Research (SDC), Niels Jensens Vej 2, DK-8000 Aarhus C, Denmark [3]Department of Chemistry, University of Florence, via della Lastruccia 3-13, I-50019, Sesto F.no, Italy and [4]Department of Physics, Chemistry and Pharmacy, University of Southern Denmark, Campusvej 55, DK-5230 Odense M, Denmark

Email: Mogens Brøndsted Nielsen - mbn@kiku.dk

*Corresponding author

1D and 2D NMR spectra of all new compounds. Table of bond lengths for VHF 2 (X-ray crystallographic data). Exponential fit of the decay over time of the VHF 7 absorbance at the longest-wavelength absorption maximum.

Content

NMR spectra of compounds **8**, **10**, **12**, **14**, **15**, **17**, **19**, **22** S2

X-ray crystallographic data for VHF **2** S14

UV–vis absorption spectra of DHA **8** and VHF **7** S15

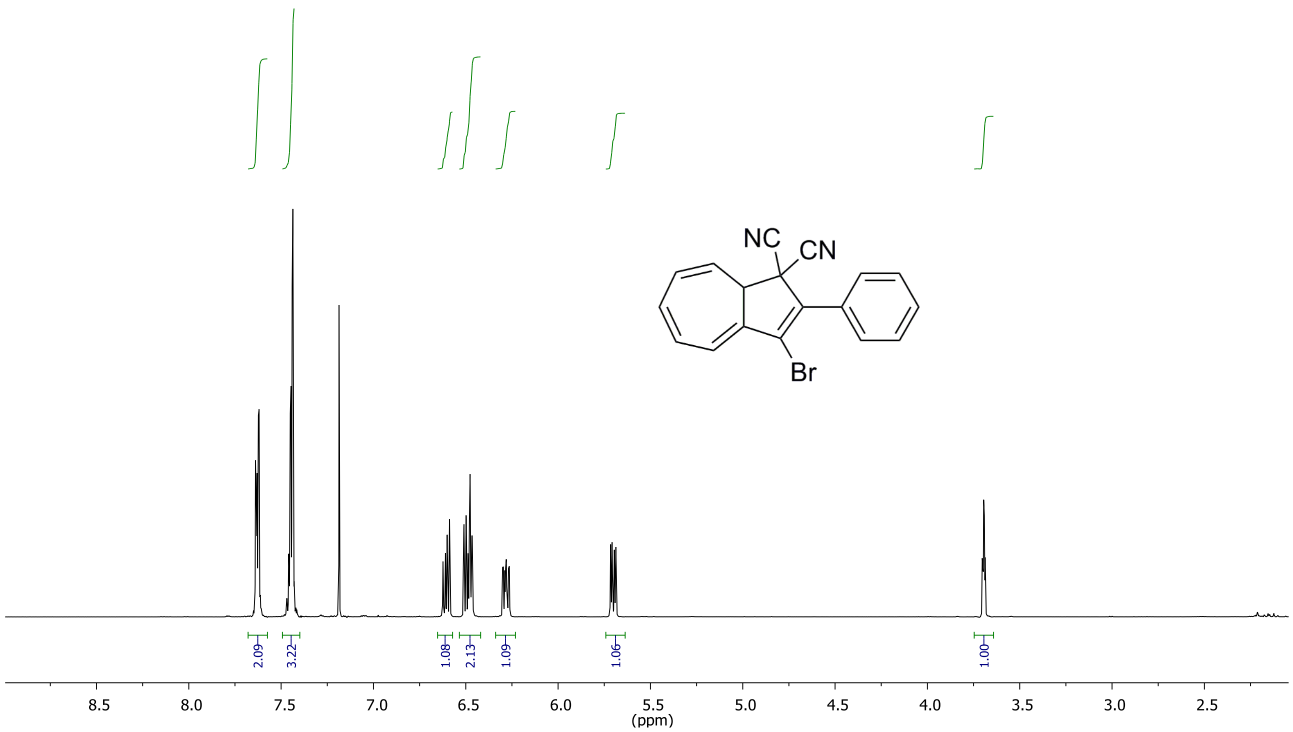

Figure S1: Compound **8**, ^{1}H NMR (500 MHz, $CDCl_3$).

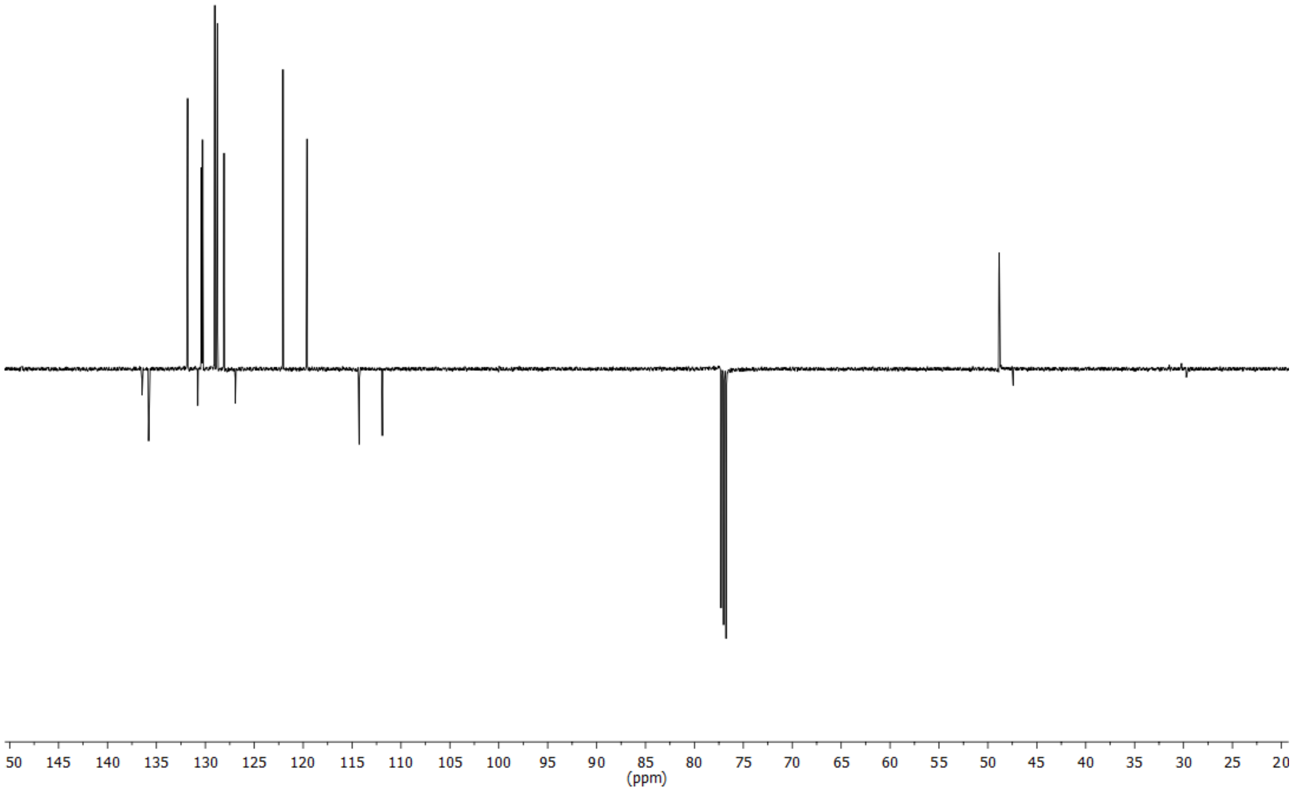

Figure S2: Compound **8**, APT (125 MHz, $CDCl_3$).

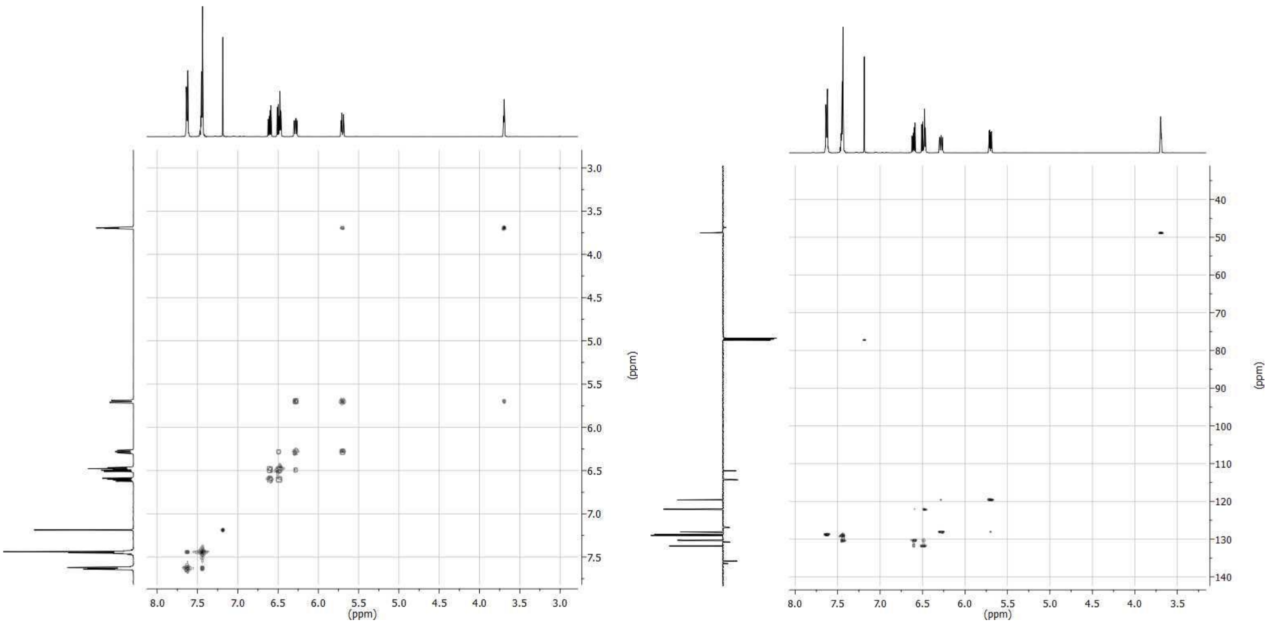

Figure S3: Compound **8**; left: COSY ($CDCl_3$), right: HSQC ($CDCl_3$).

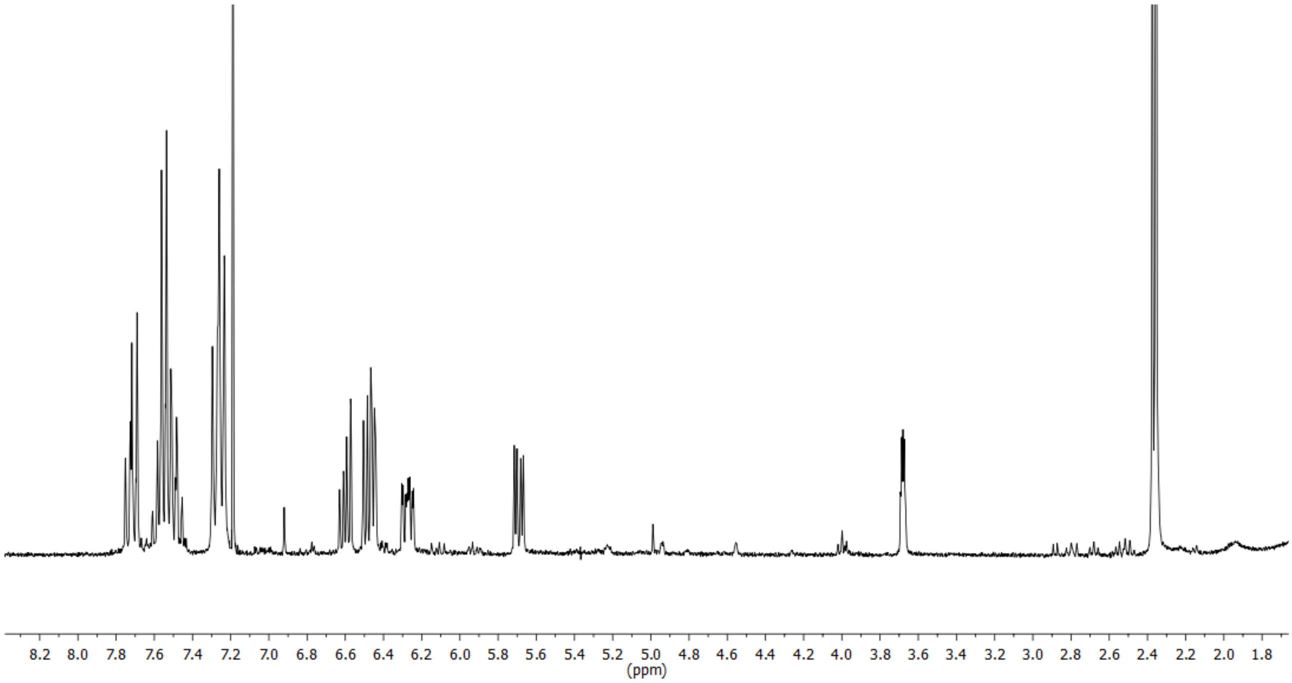

Figure S4: Compound **10**; ^{1}H NMR with minor impurities (300 MHz, $CDCl_3$).

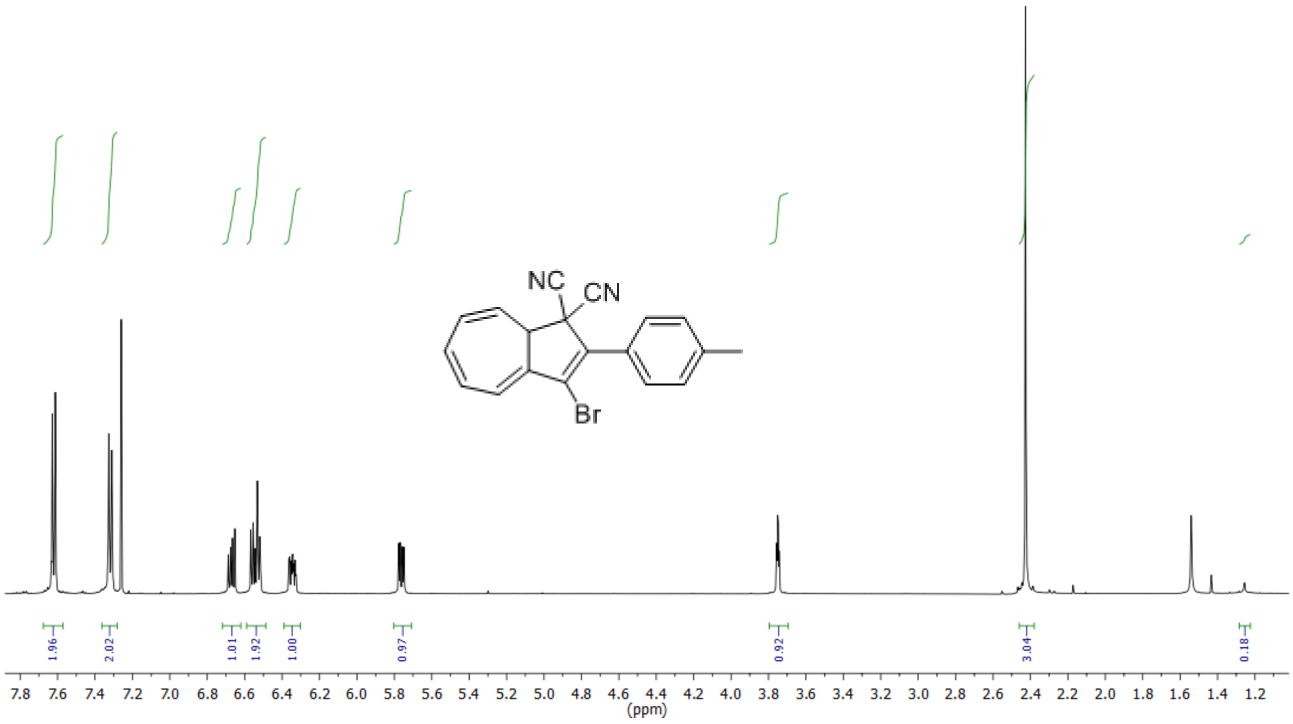

Figure S5: Compound **10**; ^{1}H NMR of pure compound (500 MHz, $CDCl_3$)

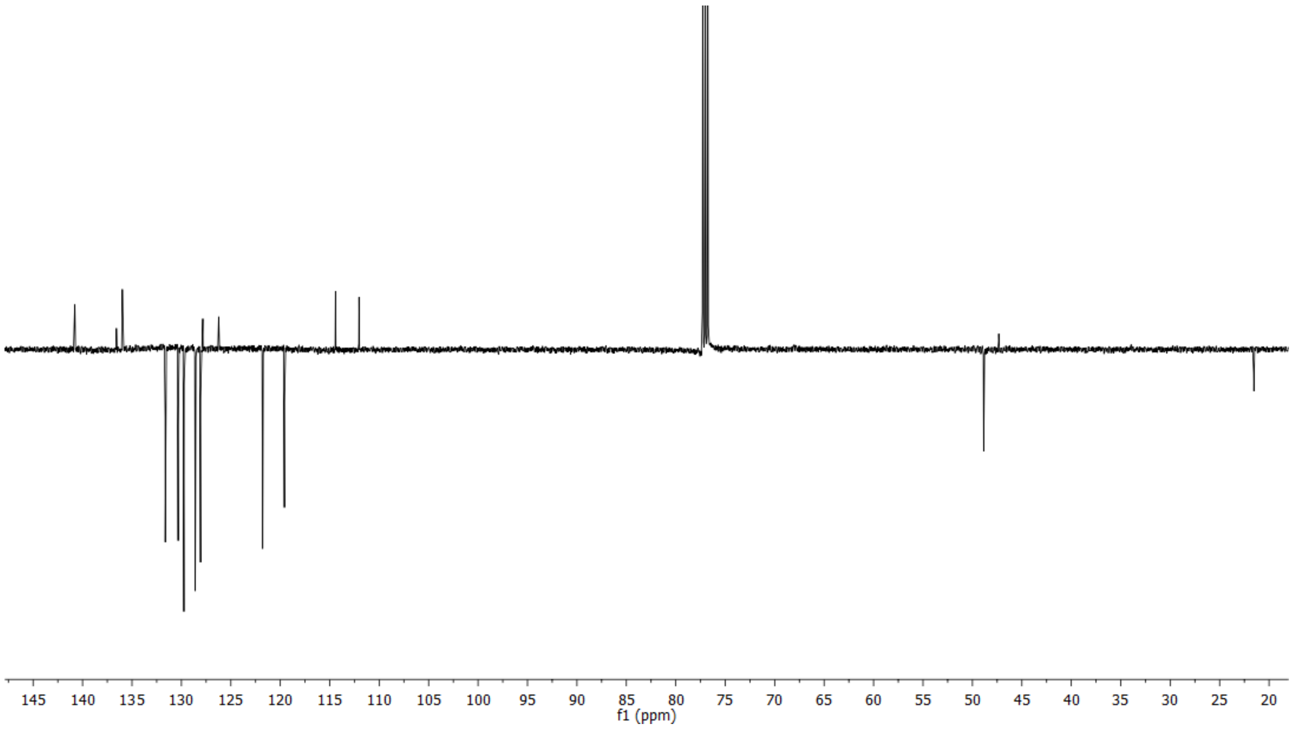

Figure S6: Compound **10**; APT (125 MHz, $CDCl_3$)

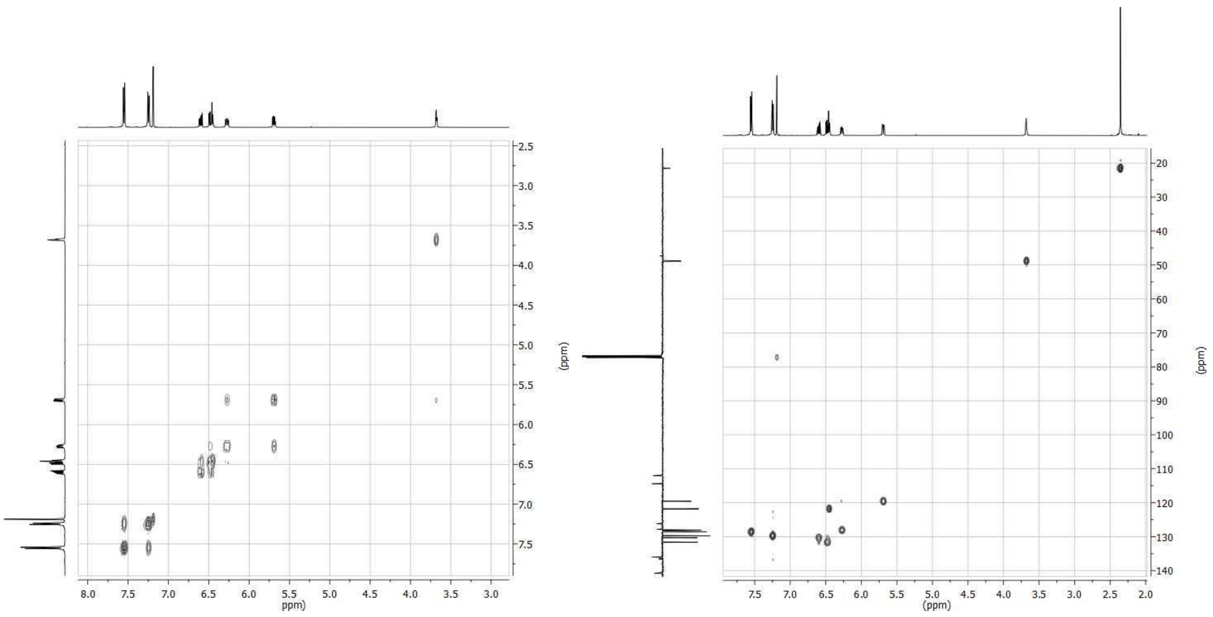

Figure S7: Compound **10**; COSY (left) and HSQC (right) in $CDCl_3$.

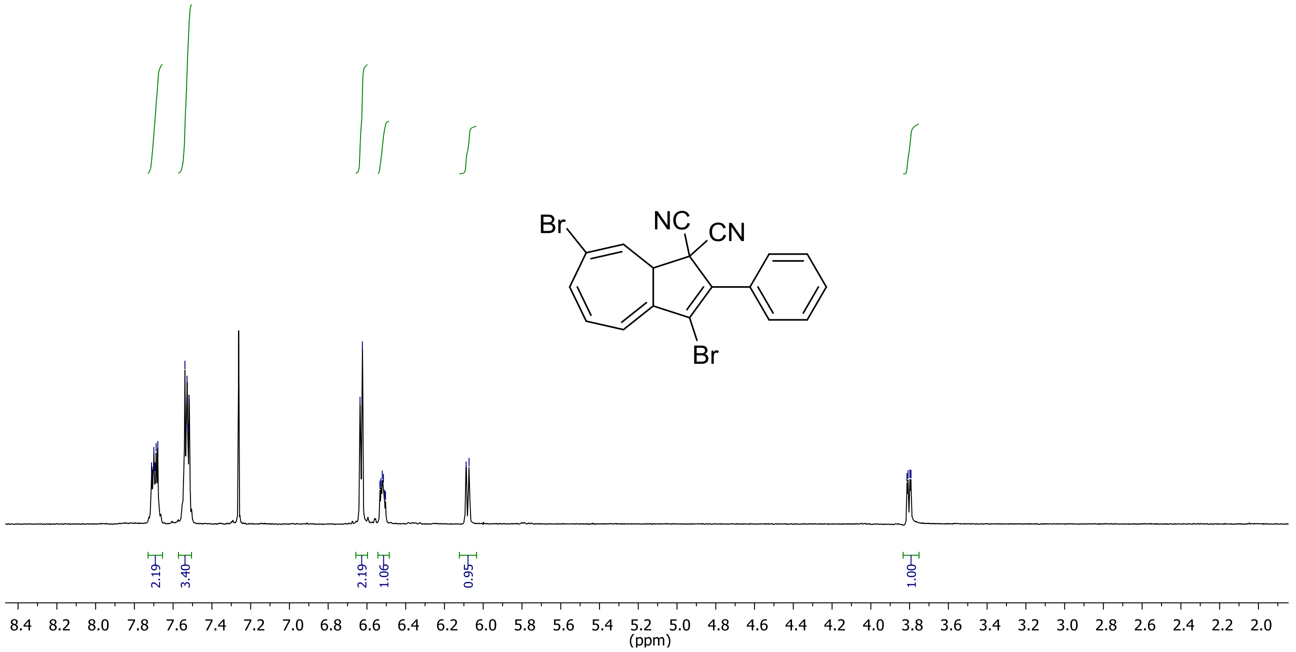

Figure S8: Compound **12**; ^{1}H NMR (300 MHz, $CDCl_3$).

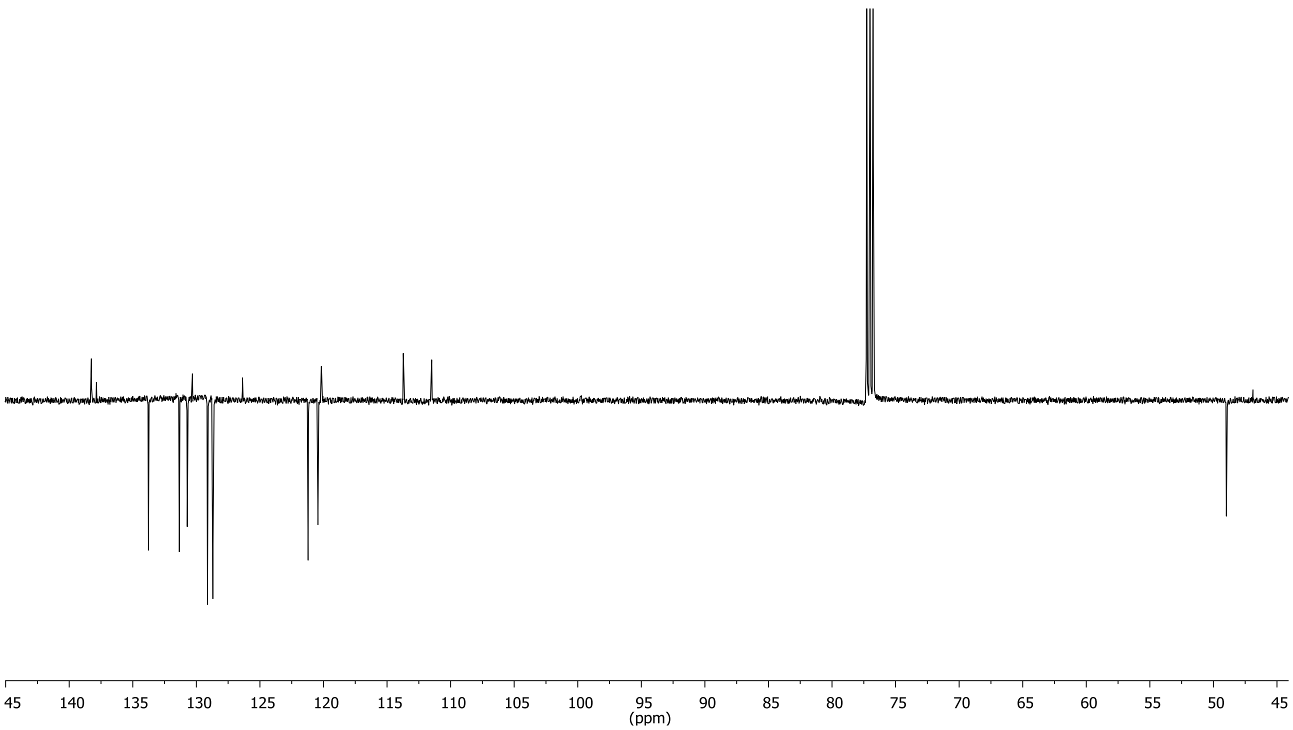

Figure S9: Compound **12**; APT (125 MHz, $CDCl_3$).

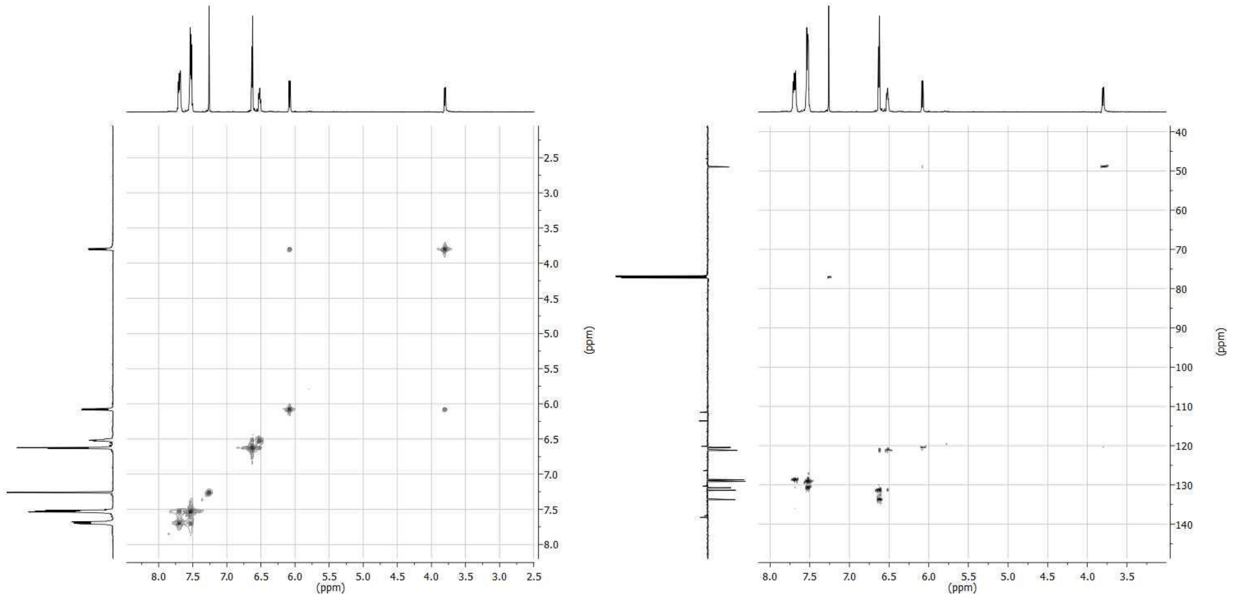

Figure S10: Compound **12**; left: COSY ($CDCl_3$), right: HSQC ($CDCl_3$).

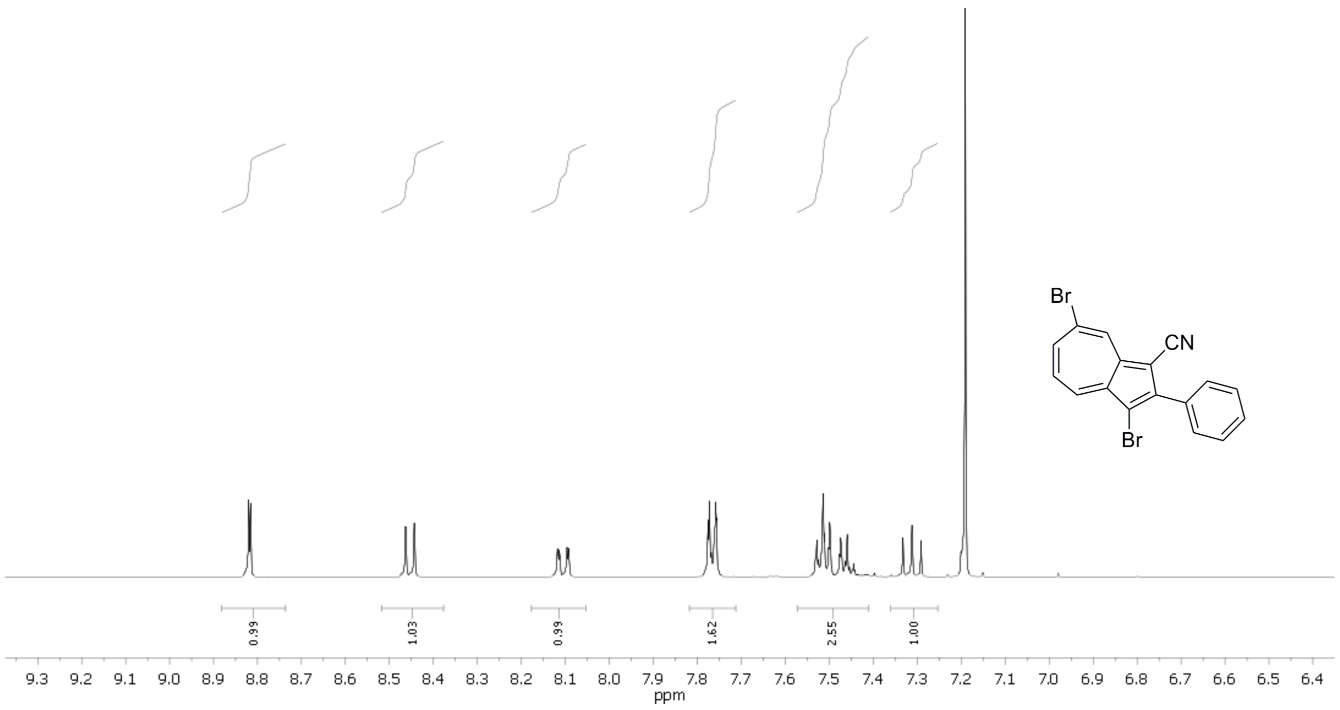

Figure S11: Compound **14**; ^{1}H NMR (500 MHz, $CDCl_3$).

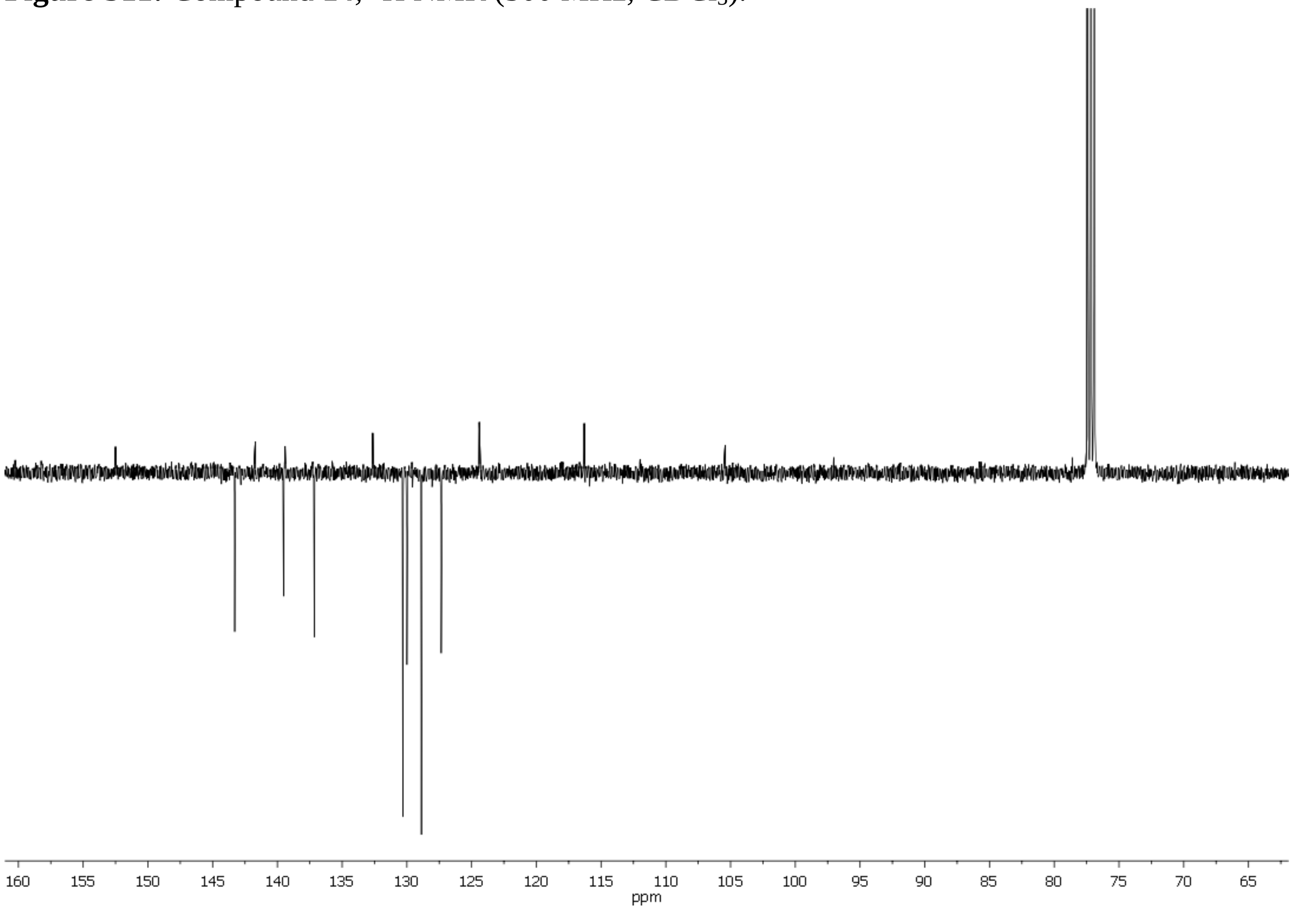

Figure S12: Compound **14**; APT (125 MHz, $CDCl_3$).

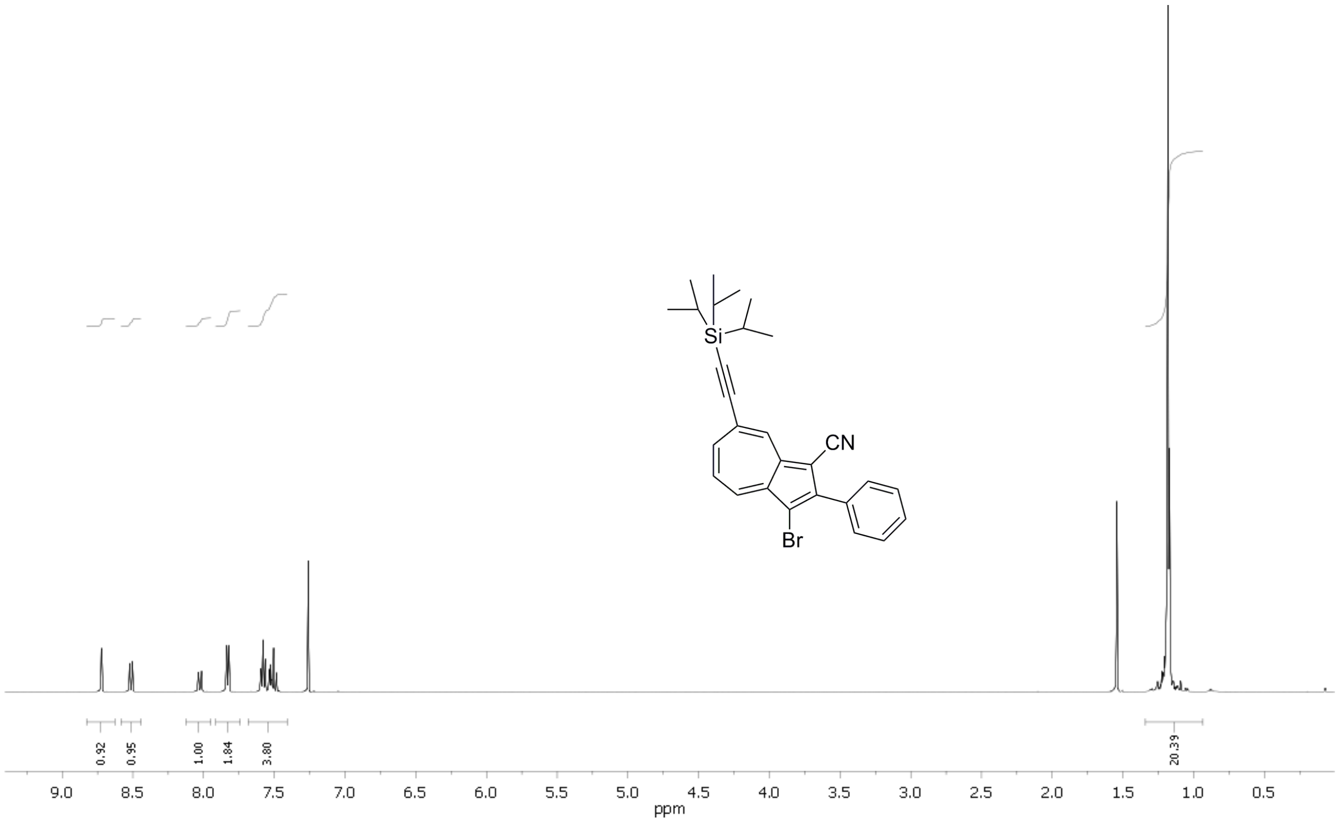

Figure S13: Compound **15**; ^{1}H NMR (500 MHz, $CDCl_3$).

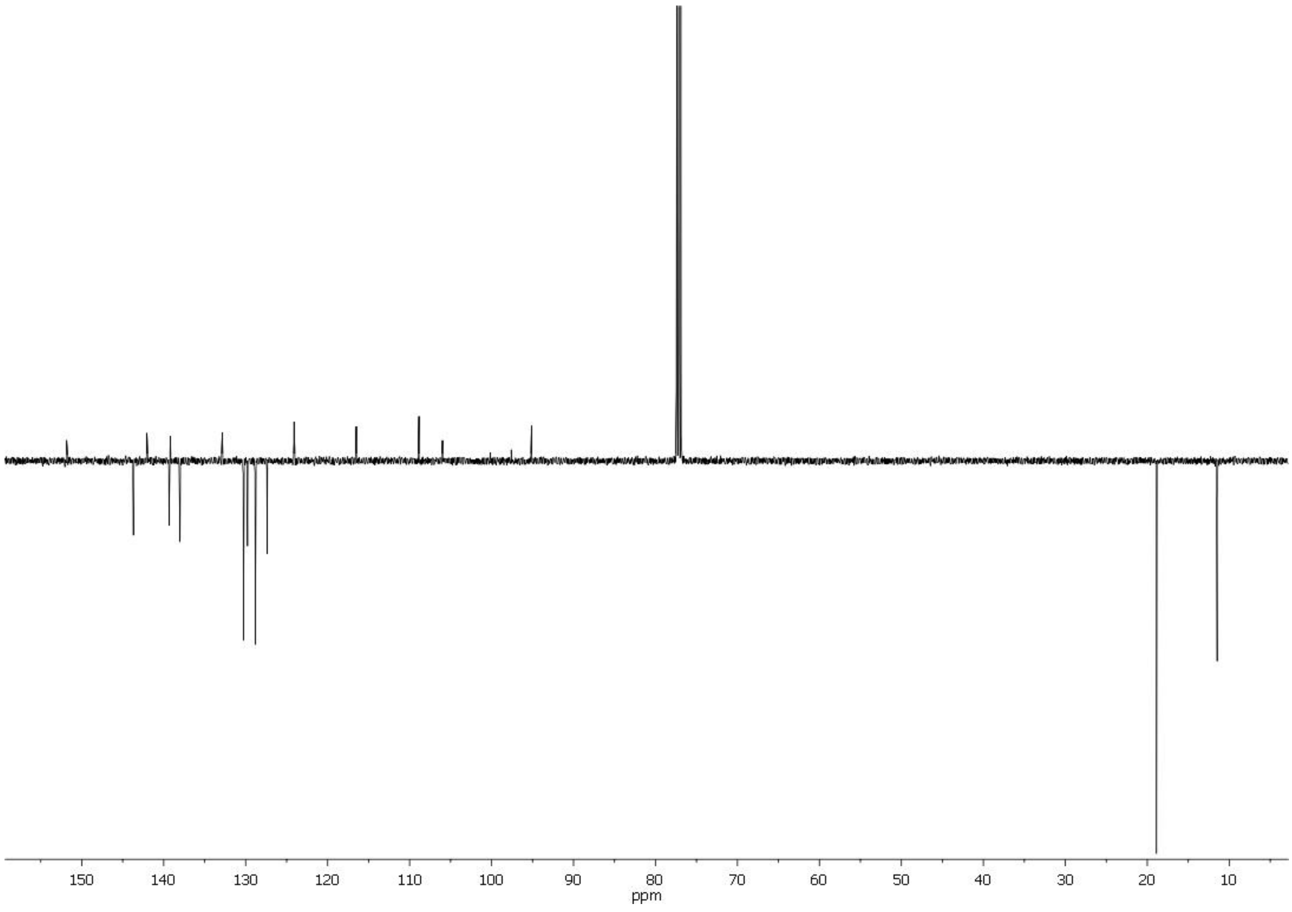

Figure S14: Compound **15**; APT (125 MHz, $CDCl_3$).

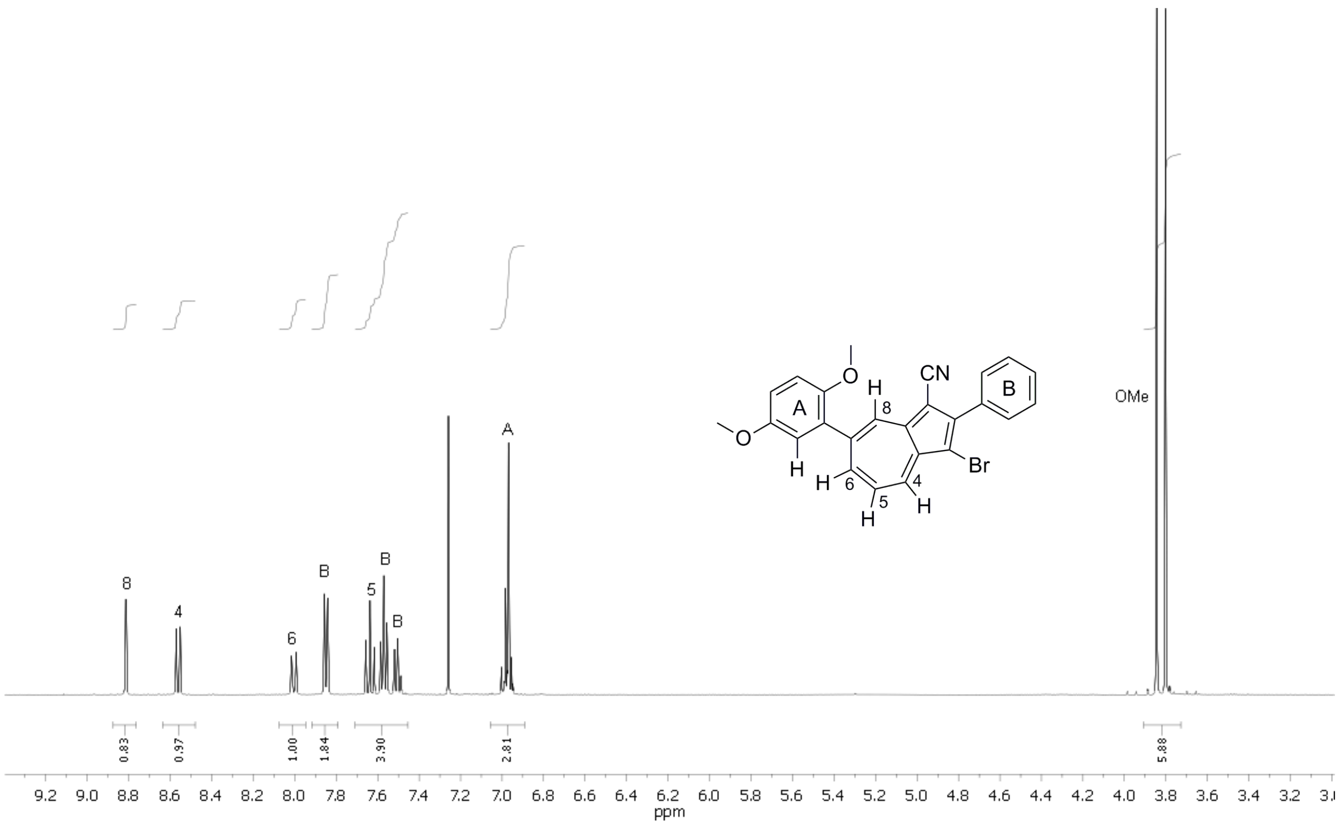

Figure S15: Compound **17**; ^{1}H NMR (500 MHz, $CDCl_3$).

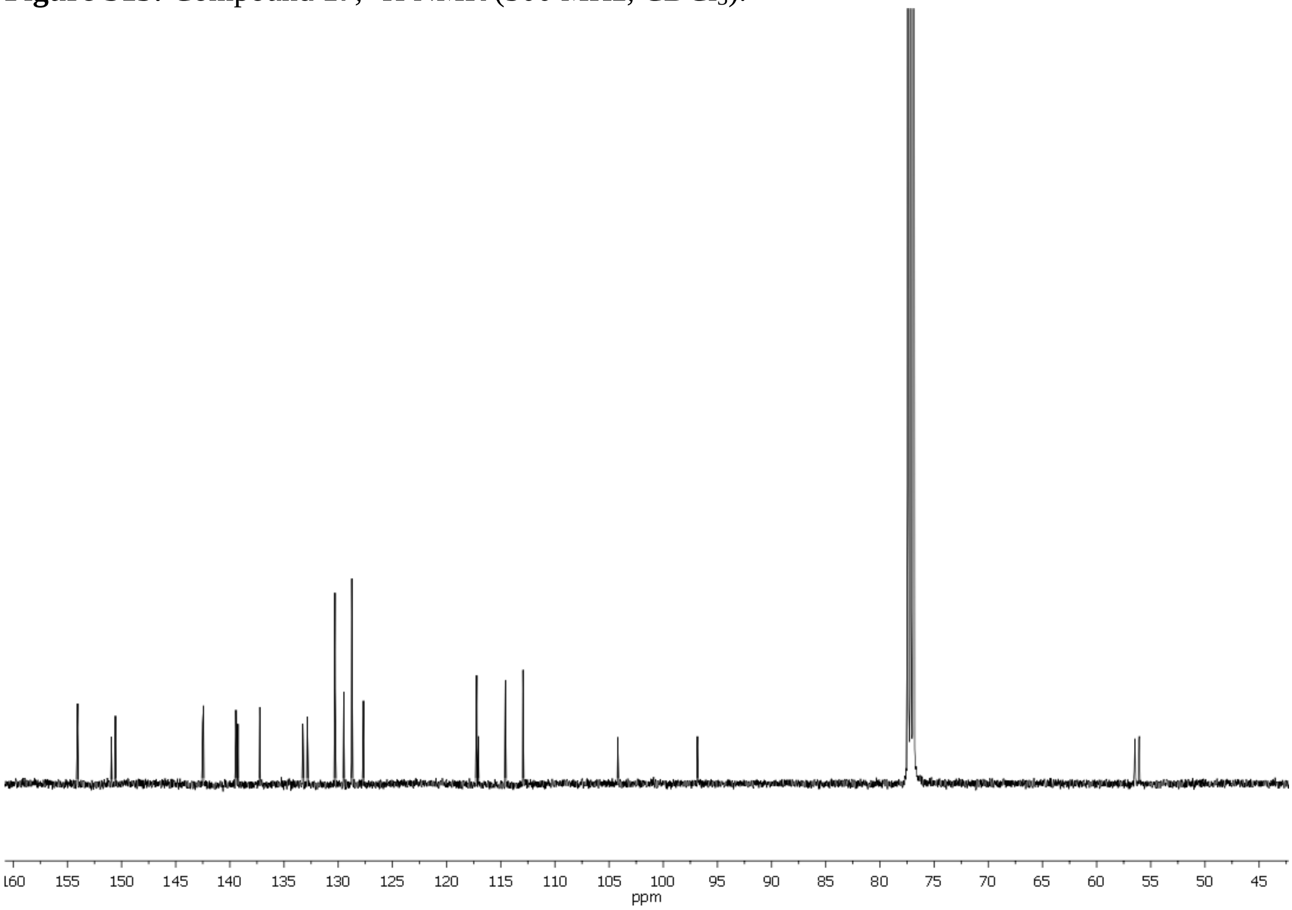

Figure S16: Compound **17**; ^{13}C NMR (125 MHz, $CDCl_3$).

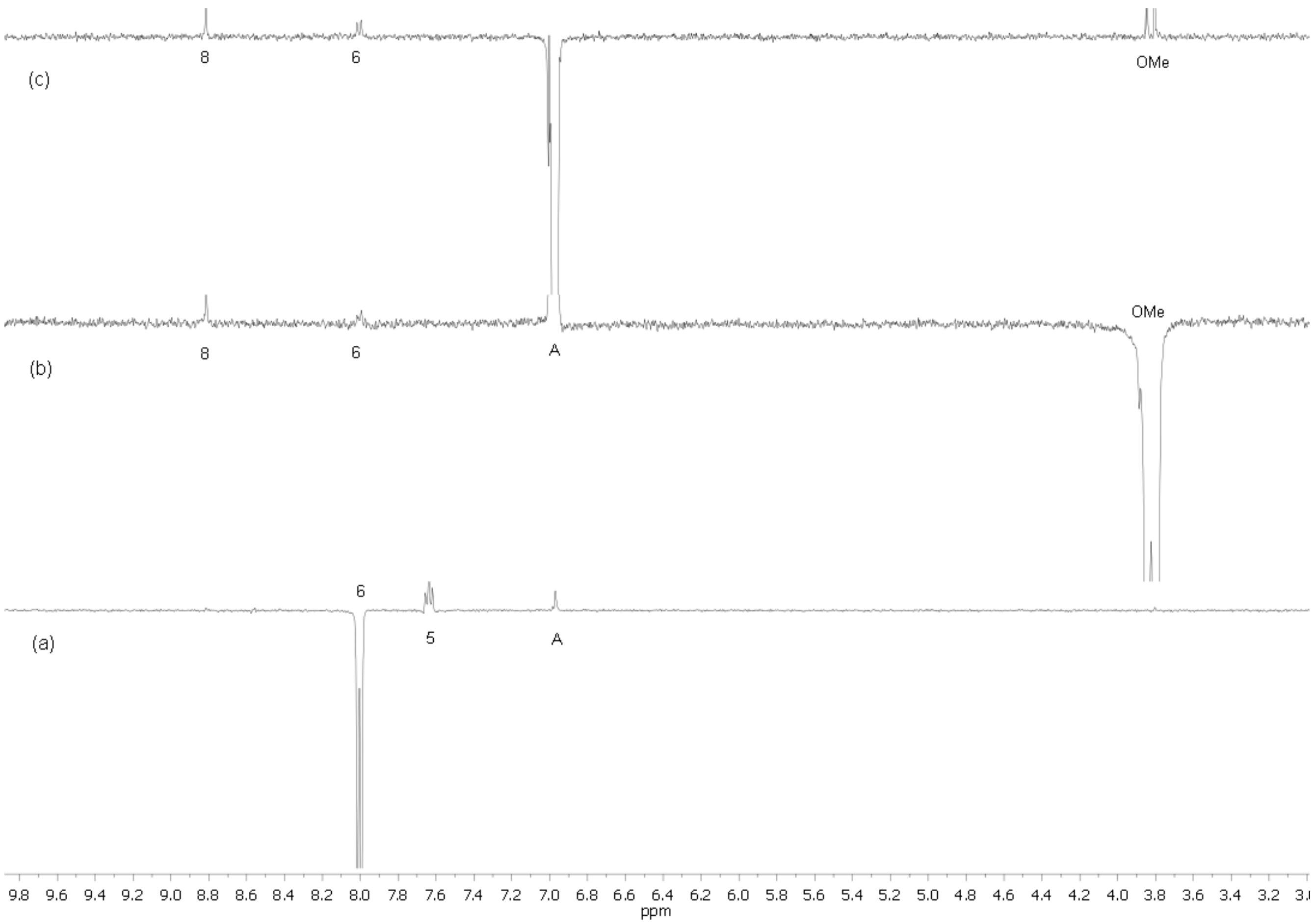

Figure S17: NOESY1D (500 MHz, $CDCl_3$) of compound **17** with irradiation on H-6 (a), OMe (b) and on the aromatic protons of ring A (c).

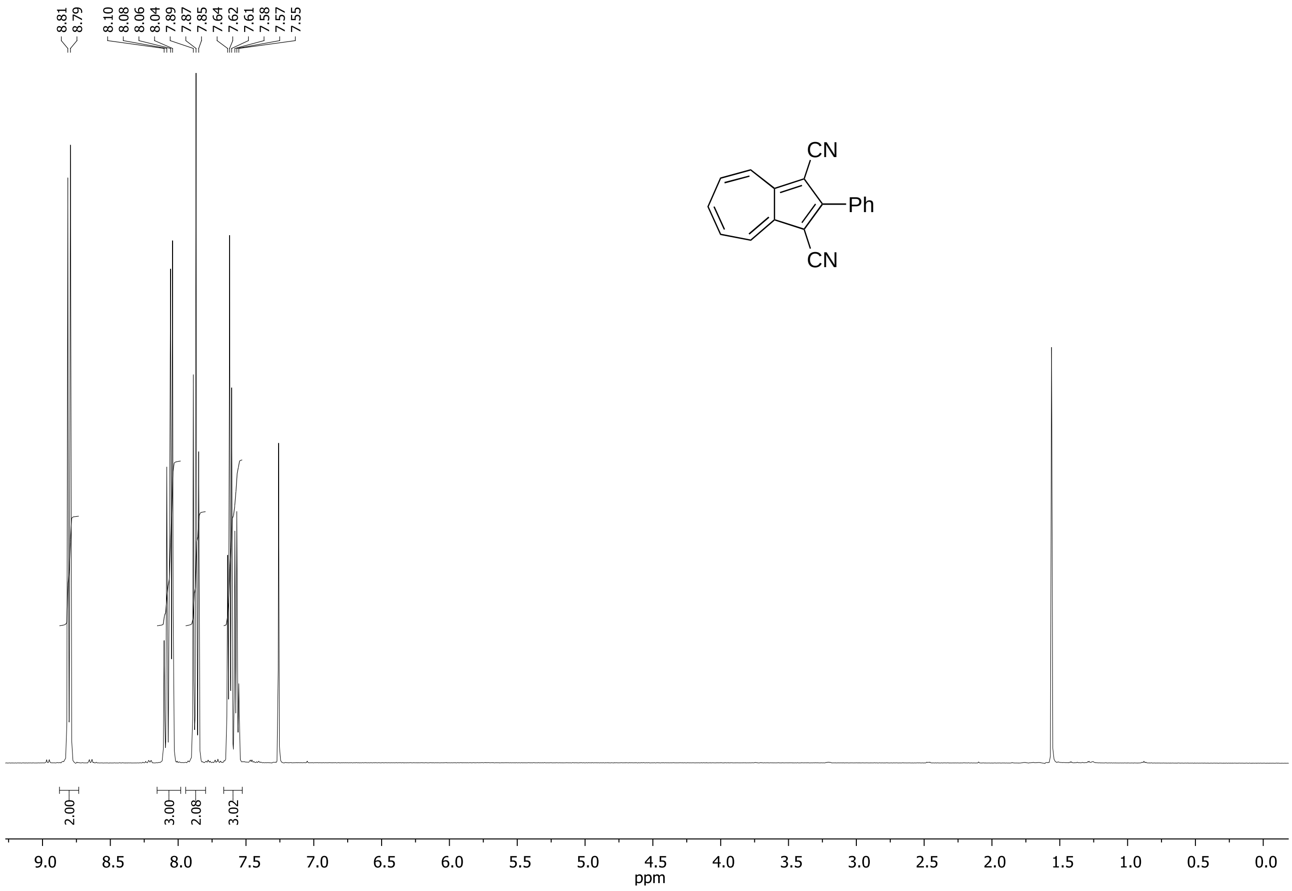

Figure S18: Compound **19**; ^{1}H NMR (500 MHz, $CDCl_3$).

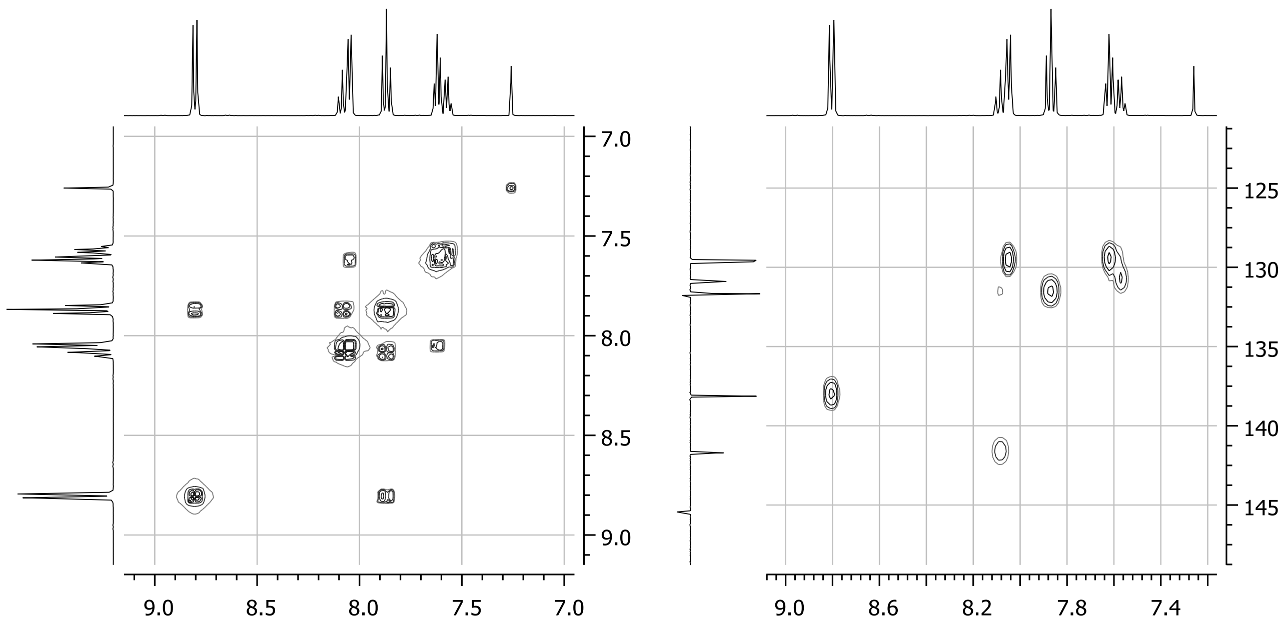

Figure S19: Compound **19** left: COSY ($CDCl_3$), right: HSQC ($CDCl_3$).

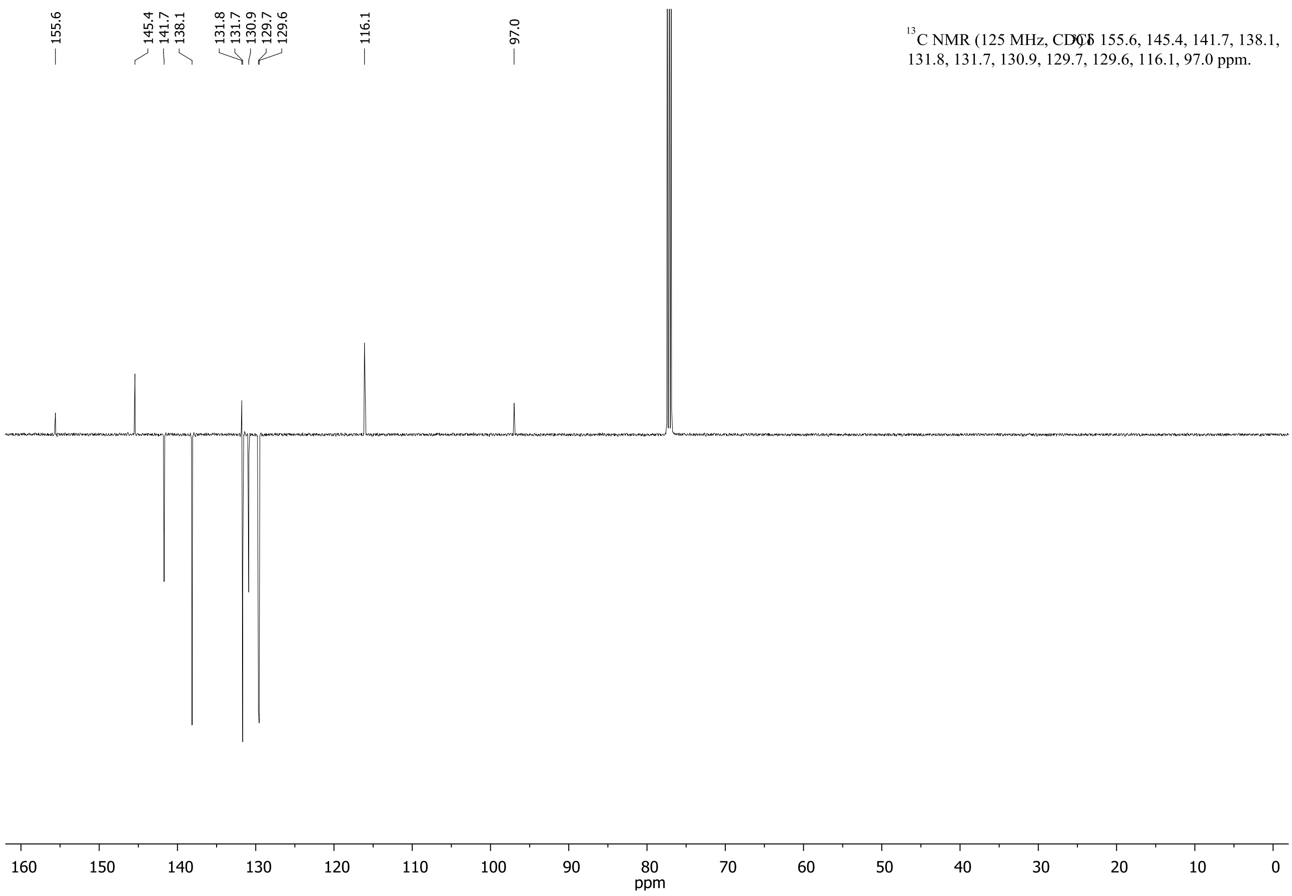

Figure S20: Compound **19**; ^{13}C NMR (125 MHz, $CDCl_3$).

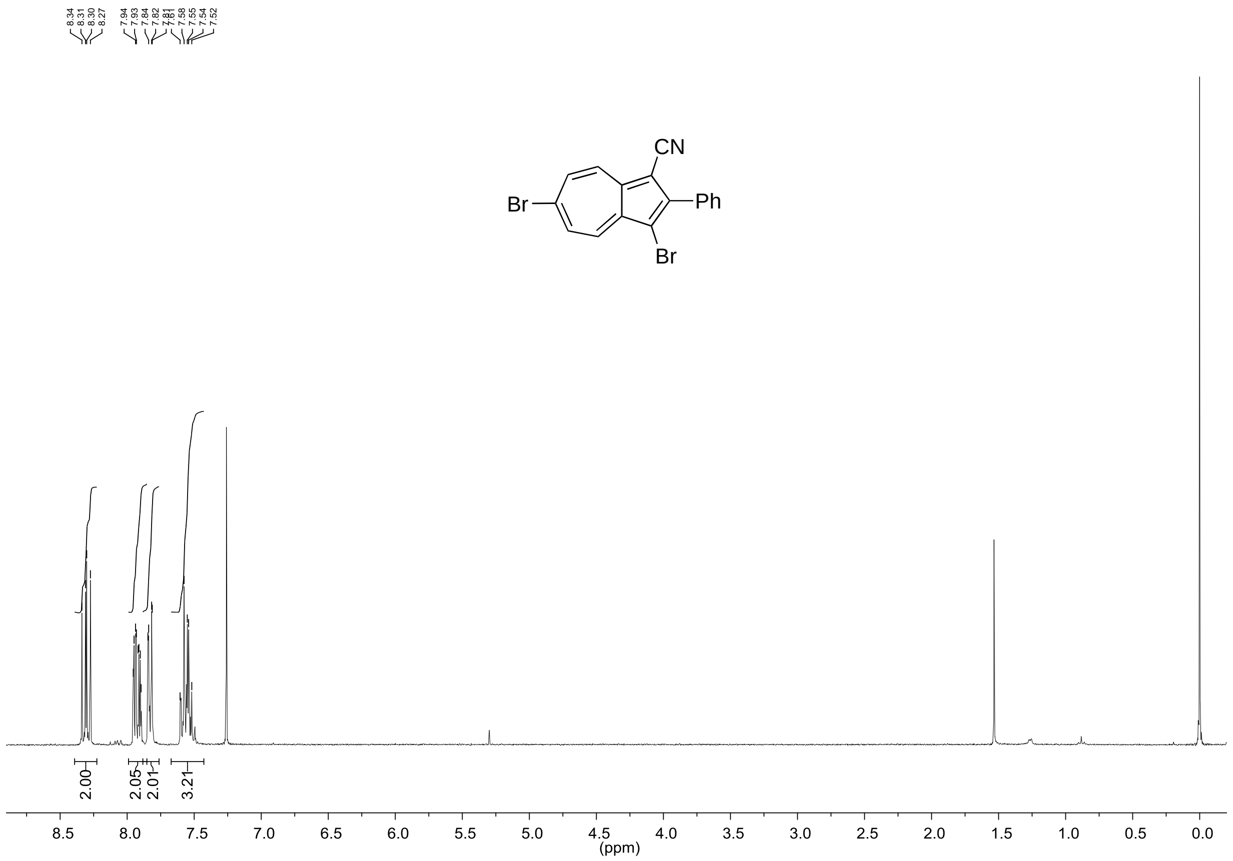

Figure S21: Compound **22**; ^{1}H NMR (300 MHz, $CDCl_3$).

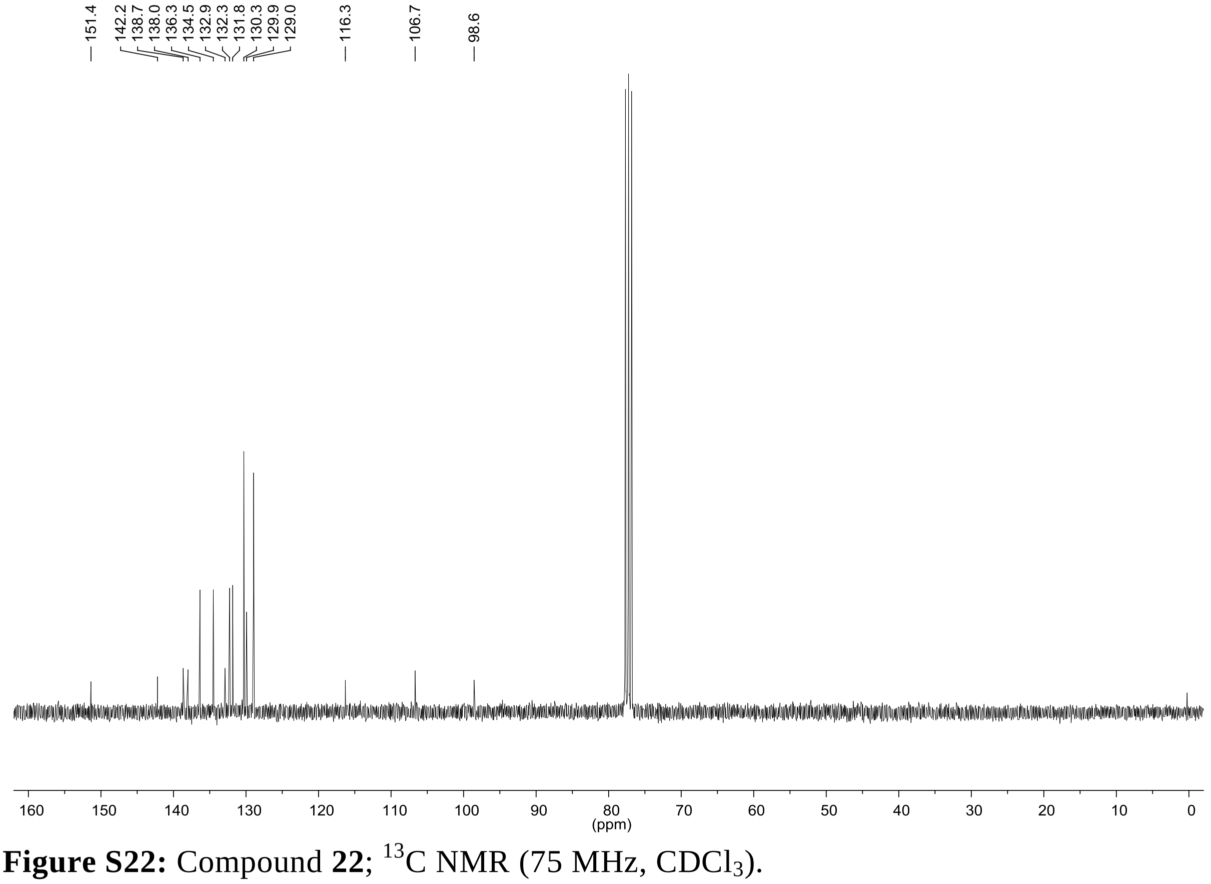

Figure S22: Compound **22**; ^{13}C NMR (75 MHz, $CDCl_3$).

X-Ray Crystallography – VHF 2

Single-crystal X-ray diffraction data were collected at 122 K by using a Nonius Kappa CCD area-detector diffractometer with Mo Kα radiation (λ = 0.71073 Å) equipped with an Oxford Cryostreams low-temperature device. The structure was solved by using direct methods (SHELXS97) and refined with the SHELXL97 software package [1]. All nonhydrogen atoms were refined anisotropically. All hydrogen atoms were located in the difference Fourier map but refined at the calculated positions. Supplementary crystallographic data (CCDC-866016) can be obtained free of charge from The Cambridge Crystallographic Data Centre via www.ccdc.cam.ac.uk/data_request/cif.

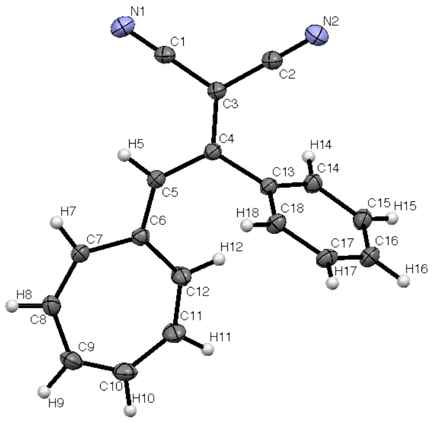

Table 1: Bond lengths (Å) (VHF **2**).

N1—C1	1.1476 (15)
N2—C2	1.1561 (16)
C1—C3	1.4366 (15)
C2—C3	1.4289 (16)
C3—C4	1.3840 (15)
C4—C5	1.4276 (15)
C4—C13	1.4898 (14)
C5—C6	1.3918 (15)
C6—C12	1.4452 (15)
C6—C7	1.4461 (16)
C7—C8	1.3591 (16)
C8—C9	1.4318 (18)
C9—C10	1.351 (2)
C10—C11	1.4247 (19)
C11—C12	1.3626 (16)
C13—C18	1.3982 (15)
C13—C14	1.3990 (16)
C14—C15	1.3917 (15)
C15—C16	1.3929 (17)
C16—C17	1.3892 (18)
C17—C18	1.3915 (15)

Table 2: Crystallographic data (VHF **2**).

Compound reference	CCDC-866016
Chemical formula	$C_{18}H_{12}N_2 \cdot 0.5(C_6H_6)$
Formula Mass	295.35
Crystal system	Monoclinic
a/Å	16.8614 (9)
b/Å	6.2465 (11)
c/Å	30.679 (3)
α/°	90.00
β/°	96.345 (11)
γ/°	90.00
Unit cell volume/Å^3	3211.4 (7)
Temperature/K	122(1)
Space group	$C2/c$
No. of formula units per unit cell, Z	8
Radiation type	Mo Kα
Absorption coefficient, μ/mm^{-1}	0.072
No. of reflections measured	47476
No. of independent reflections	7621
R_{int}	0.0560
Final R_1 values ($I > 2\sigma(I)$) [a]	0.0644
Final $wR(F^2)$ values ($I > 2\sigma(I)$) [b]	0.1522
Final R_1 values (all data) [a]	0.0901
Final $wR(F^2)$ values (all data) [b]	0.1669
Goodness of fit on F^2	1.110

[a] $R_1 = \Sigma||F_o| - |F_c||/\Sigma|F_o|$. [b] $wR = [\Sigma w(F_o^2 - F_c^2)^2/\Sigma w(F_o^2)^2]^{1/2}$

Absorption spectra

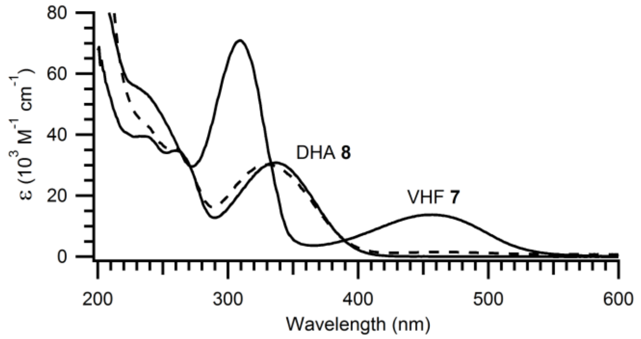

Figure S23: Absorption spectra of DHA **8** and VHF **7** in cyclohexane. The broken curve shows the absorption spectrum after one light–heat cycle (DHA → VHF → DHA).

Thermal conversion of VHF 7 to DHA 8

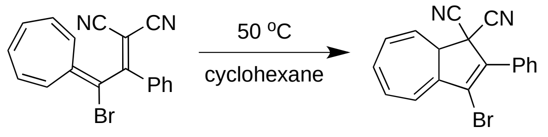

7 **8**

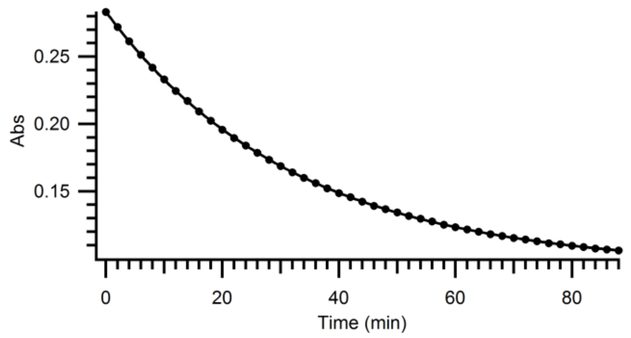

Figure S24: The decay in the VHF absorption maximum at 453 nm over time was fitted by an exponential function (first-order kinetics). Solvent, cyclohexane; temperature, 50 °C.

References

[1] Sheldrick, G. M., *Acta Cryst.*, **2008**, *A64*, 112-122.
